# Glioma-Associated Microglia/Macrophages Display an Expression Profile Different from M1 and M2 Polarization and Highly Express *Gpnmb* and *Spp1*

**DOI:** 10.1371/journal.pone.0116644

**Published:** 2015-02-06

**Authors:** Frank Szulzewsky, Andreas Pelz, Xi Feng, Michael Synowitz, Darko Markovic, Thomas Langmann, Inge R. Holtman, Xi Wang, Bart J. L. Eggen, Hendrikus W. G. M. Boddeke, Dolores Hambardzumyan, Susanne A. Wolf, Helmut Kettenmann

**Affiliations:** 1 Max-Delbrueck-Center for Molecular Medicine, Berlin, Germany; 2 Department of Experimental Neurology, Charité–University Medicine Berlin, Berlin, Germany; 3 Department of Neurosciences, Cleveland Clinic, Cleveland, Ohio, United States of America; 4 Department of Molecular Medicine, Cleveland Clinic Lerner College of Medicine, Case Western Reserve University, Cleveland, Ohio, United States of America; 5 Department of Neurosurgery, Charité –Universitätsmedizin Berlin, Berlin, Germany; 6 Department of Neurosurgery, Helios Clinics, Berlin, Germany; 7 Department of Ophthalmology, University of Cologne, Cologne, Germany; 8 Department of Neuroscience, Section Medical Physiology, University of Groningen, University Medical Center Groningen, Groningen, The Netherlands; University of Florida, UNITED STATES

## Abstract

Malignant glioma belong to the most aggressive neoplasms in humans with no successful treatment available. Patients suffering from glioblastoma multiforme (GBM), the highest-grade glioma, have an average survival time of only around one year after diagnosis. Both microglia and peripheral macrophages/monocytes accumulate within and around glioma, but fail to exert effective anti-tumor activity and even support tumor growth. Here we use microarray analysis to compare the expression profiles of glioma-associated microglia/macrophages and naive control cells. Samples were generated from CD11b^+^ MACS-isolated cells from naïve and GL261-implanted C57BL/6 mouse brains. Around 1000 genes were more than 2-fold up- or downregulated in glioma-associated microglia/macrophages when compared to control cells. A comparison with published data sets of M1, M2a,b,c-polarized macrophages revealed a gene expression pattern that has only partial overlap with any of the M1 or M2 gene expression patterns. Samples for the qRT-PCR validation of selected M1 and M2a,b,c-specific genes were generated from two different glioma mouse models and isolated by flow cytometry to distinguish between resident microglia and invading macrophages. We confirmed in both models the unique glioma-associated microglia/macrophage phenotype including a mixture of M1 and M2a,b,c-specific genes. To validate the expression of these genes in human we MACS-isolated CD11b^+^ microglia/macrophages from GBM, lower grade brain tumors and control specimens. Apart from the M1/M2 gene analysis, we demonstrate that the expression of *Gpnmb* and *Spp1* is highly upregulated in both murine and human glioma-associated microglia/macrophages. High expression of these genes has been associated with poor prognosis in human GBM, as indicated by patient survival data linked to gene expression data. We also show that microglia/macrophages are the predominant source of these transcripts in murine and human GBM. Our findings provide new potential targets for future anti-glioma therapy.

## Introduction

Malignant glioma (WHO grade III and IV), such as glioblastoma multiforme (GBM), are highly aggressive and account for almost 50% of all brain neoplasms. As of today no successful treatment exists, offering glioma patients an average survival time of about one year after diagnosis, despite surgical tumor resection, radio-, and chemotherapy [1]. Glioma are highly invasive and the cellular and genetic inter- and intra-tumor heterogeneity has lead to the failure of current anti-glioma therapy [2,3,4,5].

Among other immune cells, brain-resident microglia and peripheral macrophages/monocytes are attracted toward glioma in large numbers and they can amount up to 30% of the cells in the tumor tissue [6,7,8]. Experimental findings by us and others show that a tumoricidal activity of glioma-associated microglia/macrophages (GAMs) is counteracted by the glioma cells, which reprogram GAMs into tumor supportive cells. GAMs actively support tumor growth, e.g. by secreting matrix-degrading enzymes as well as immunosuppressive factors [9,10,11,12]. Thus, these stromal cells might serve as an attractive target for anti-glioma therapy and the identification of factors produced by GAMs might help to better understand disease progression [11,13,14,15,16].

Based on *in vitro* activation states macrophage polarization is divided into classical inflammatory (M1) activation (responsible for Th1 responses, type I inflammation, killing of intracellular pathogens) and alternative (M2) activation. Alternative activation can be further divided into M2a (Th2 responses, type II inflammation, killing of pathogens, allergy), M2b (Th2 activation, immunoregulation), and M2c (immunoregulation, matrix deposition, tissue remodeling) activation [17]. Although the definition of these pure activation states is based on defined *in vitro* conditions, and macrophage activation is likely to be much more diverse and complex in the *in vivo* setting, several studies have addressed the expression of polarization marker genes in GAMs, either *in vitro* or *in vivo* [9,13,18,19]. Similar to solid tumors in other organs, GAMs produce factors associated with an alternative macrophage activation, such as increased production of anti-inflammatory molecules (e.g. TGF-β1, ARG1, and IL-10), and molecules supporting tissue remodeling and angiogenesis (e.g. VEGF, MMP2, MMP9, and MT1-MMP). However, GAMs also produce pro-inflammatory molecules (e.g. TNF-α, IL1-β, and CXCL10) [9,10,11,18,20,21]. To date no comprehensive comparison of GAMs expression pattern with M1, M2a,b,c macrophages has been performed. In this study, we isolated CD11b^+^ cells from GL261 mouse gliomas and naïve control mice and performed a genome-wide microarray expression analysis. We compared the GAMs expression profile to datasets of M1, M2a, M2b, and M2c *in vitro*-stimulated macrophages. Using flow cytometry to distinguish between resident microglia and invading macrophages/monocytes, we investigated the expression of selected genes in resident and invading GAMs in two different glioma mouse models by qRT-PCR. To show the relevance for potential therapeutic applications, we investigated some key genes in CD11b^+^ GAMs isolated from human GBM samples compared to cells isolated from lower grade brain tumors, control microglia, and CD11b^-^ cells in GBM. Last, we used the The Cancer Genome Atlas (TCGA) database for glioma-patient survival data linked to gene expression data.

## Materials and Methods

### Ethics statement

This study was carried out in strict accordance with the recommendations in the Guide for the Care and Use of Laboratory Animals of the National Institutes of Health. This study was approved by the local ethics committees for animal experiments: the Institutional Animal Care and Use Committees of Cleveland Clinic, Lerner Research Institute – approved protocol number 2013-1029 (LRI, last approved June 25 2013) and the Committee on the Ethics of Animal Experiments in Berlin (LaGeSo, Permit Numbers: GO 268-10, GO 343-10, GO 438-12). All surgery was performed under anesthesia, and all efforts were made to minimize suffering.

Freshly resected patient samples were provided by the Department for Neurosurgery Charité University Hospital and the Helios Clinics (both Berlin, Germany). Handling and analysis of these tissues was performed according to the rules and with the approval of the Ethical Committee and with patient’s written consents (Ethics Committee: “Ethikkommission der Charité—Universitätsmedizin Berlin”, application number: EA4/098/11).

### Animals

All *in vivo* work with GL261 glioma cells was done in C57BL/6 wild-type mice (Charles River Laboratories, Wilmington, MA, USA). Age matched naïve C57BL/6 wild-type mice were used as controls.

To broaden the relevance of our findings, we employed another murine model where the tumor is initiated by the overexpression of PDGFb in *Nestin*-expressing cells *in vivo*: *Ntv-a*/*Ink4a-Arf^-/-^* mice develop pro-neural high-grade gliomas 6 to 8 weeks following intracranial injection of RCAS-PDGFb-producing DF-1 chicken fibroblast cells at 4.5 to 10 weeks of age [22]. The genetic backgrounds of tv-a mice are FVB/N, C57BL/6, BALB/C, and tv-a. RCAS-PDGFb tumors were dissociated as described below and intracranially re-transplanted into *Cx3cr1* ^*GFP/wt*^ *Ccr2* ^*RFP/wt*^ mice (kind gift of Richard Ransohoff, [23]) to distinguish between microglia and peripheral monocytes that invaded the brain. Naïve age-matched *Cx3cr1* ^*GFP/wt*^ *Ccr2* ^*RFP/wt*^ mice were used as controls. 5 to 7 weeks after intracranial injection of RCAS tumor cells into *Cx3cr1* ^*GFP/wt*^ *Ccr2* ^*RFP/wt*^ mice these mice develop tumors that are histologically identical to the original tumors in *Ntv-a*/*Ink4a-Arf^-/-^* mice.

### Generation of intracranial mouse gliomas

Injections were performed using a stereotactic frame (Stoelting, Wood Dale, IL, USA). Mice used for these experiments were 8 to 10-week-old (C57BL/6 mice for GL261 injection), 4.5 to 10-week-old (*Ntv-a*/*Ink4a-Arf^-/-^* mice for DF-1 RCAS-PDGFb injection), or 6–10-week-old (*Cx3cr1* ^*GFP/wt*^ *Ccr2* ^*RFP/wt*^ mice for RCAS-PDGFb tumor cell re-implantation). Mice were anesthetized with intraperitoneal injections of ketamine (0.1 mg/g, Pharmazeutischen Handelsgesellschaft, Garbsen, Germany) and xylazine (0.02 mg/g, Bayer, Leverkusen, Germany). Animals were also provided 0.25% Marcaine in the volume of about 0.1ml/25g administered right before the surgery, which provided pain relief from the sutures for 6–8 hours.

One microliter cell suspension (2x10^4^ GL261 cells, 4×10^4^ transfected DF-1 cells, or 5x10^4^ RCAS-PDGFb tumor cells) was delivered using a 30-gauge needle attached to a Hamilton syringe (Hamilton, Reno, NV, USA). Coordinates for GL261 injections into C57BL/6 mice were bregma 1 mm anterior, Lat (lateral) -2 mm (right of midline), and a depth -3 mm from the dural surface. Coordinates for injections of DF-1 cells, and RCAS-PDGFb tumor cells into *Ntv-a*/*Ink4a-Arf^-/-^* mice, and *Cx3cr1* ^*GFP/wt*^ *Ccr2* ^*RFP/wt*^ mice, respectively were bregma 1.5 mm anterior, Lat -0.5 mm, and a depth 2.0 mm. Mice were monitored daily for the first two weeks and twice a day starting from day 15 post-injection for symptoms of tumor development (lethargy, hydrocephalus, head tilting). The size of resulting tumors ranged from 1.5–2.5 mm (GL261 tumors) and 2.5–3.5 mm (RCAS-PDGFb tumors).

### Cultivation of Cell Lines

Cells of the murine GL261 glioma cell line (National Cancer Institute, MD, USA) were grown in Dulbecco’s modified Eagle’s medium (DMEM) with 10% fetal calf serum (FCS), 200 mM glutamine, 100 U/mL penicillin, and 100 mg/ml streptomycin (all from Invitrogen, Carlsbad, CA, USA). DF-1 cells were purchased from ATCC (Manassas, VA, USA). Cells were grown at 39°C according to ATCC instructions. Transfections with RCAS-PDGFb were performed using Fugene 6 transfection kit (no. 11814443001; Roche, Mannheim, Germany) according to manufacturer’s protocol.

### Tumorsphere Culture

RCAS-PDGFb tumors were excised from tumor brains using a scalpel, minced, and incubated with Accutase (eBioscience, San Diego, CA, USA) for 15 minutes at 37°C. Tissue pieces were mechanically dissociated using a 1 ml pipette and washed in Dulbecco modified Eagle medium (DMEM; Sigma-Aldrich, St. Louis, MO, USA). Cells were passed through a 70 μm cell strainer and seeded into a T25 cell culture flask. Cells were grown in GIC medium containing DMEM-F12 GlutaMAX (GIBCO-Invitrogen, Carlsbad, CA, USA), 1% penicillin G/streptomycin sulfate (Sigma-Aldrich), B-27 without vitamin A (1:50; GIBCO-Invitrogen), HEPES (0.2 mM, Sigma-Aldrich), insulin (20 ng/ml, Sigma-Aldrich), supplemented with fibroblast growth factor 2 (FGF2, 20 ng/ml, Cell Systems, Kirkland, WA, USA) and epidermal growth factor (EGF, 20 ng/ml, Cell Systems).

### Human tissue

Tumor and control tissue was taken during surgery while patients were under a general anesthetic, and was placed immediately in culture medium for transportation. Cells were isolated from the tissue as described below no later than 2 hours after resection. Cell sorting was performed via MACS isolation, using anti-CD11b microbeads (described below).

### Cell Isolation

GL261-implanted mice were sacrificed 20 days post-injection, RCAS-PDGFb-implanted mice were sacrificed 4–5 weeks post-injection. Tumor-bearing and control mice were euthanized by i.p. injection of 200 μl pentobarbital-sodium (Narcoren, Pharmazeutischen Handelsgesellschaft) and perfused using a 0.9% NaCl solution. The brain and spleen were extracted and stored in ice-cold HBSS (Gibco-Invitrogen). For naïve mouse brains, the olfactory bulbs and the cerebellum were cut by a scalpel and discarded. The rest of the tissue was used for dissociation. In tumor-bearing mouse brains, only the visible tumor area around the injection site was used.

Human and mouse tissue was dissociated with the Neural Tissue Dissociation Kit (Miltenyi Biotec, Bergisch-Gladbach, Germany) according to the manufacturer’s instructions. To remove the myelin we followed a protocol published elsewhere [24]. In brief, the brain cell suspension was mixed with a total of 25 ml of a 22% Percoll (Th.Geyer, Renningen, Germany) solution and a layer of 5 ml cold PBS (Gibco-Invitrogen) was added on top. Centrifugation at 950 g with slow acceleration and without breaks created a gradient that separated the cell pellet on the bottom of the tube from the myelin which was carefully aspirated. For the isolation of GAMs from RCAS-PDGFb tumors-bearing and corresponding naïve *Cx3cr1* ^*GFP/wt*^ *Ccr2* ^*RFP/wt*^ mice a 30%/70% Percoll gradient was used. After 25 min of centrifugation at 800 g GAMs and naïve microglia were enriched at the 30%/70% interphase. Cells were collected, washed once with PBS, and subsequently centrifuged again at 300 g for 10 min. Subsequently, the cell pellet was resuspended in sorting buffer for subsequent magnetic-activated cell sorting (MACS) or fluorescence-activated cell sorting (FACS) isolation.

Spleens were processed through a 70 μm cell strainer with a syringe plunger and the mesh rinsed with 10 ml of PBS per spleen. The cells were centrifuged and the pellet subjected to erythrocyte lysis by adding 5 ml of 1x RBC lysis buffer (Cat# 420301, Biolegend, San Diego, CA, USA). The lysis was carried out by shaking the tube mildly for 5 min at RT and subsequently stopped with 20 ml of PBS. The pellet was washed once with PBS and resuspended in PBS, containing 0.5% FCS and 2mM EDTA (FACS buffer) for subsequent FACS isolation.

For RCAS-PDGFb tumors blood monocytes were used as peripheral controls (instead of monocytes isolated from spleens). To obtain blood monocytes, 200 μl of blood was mixed with 500 μl of PBS, containing 2.5 mM EDTA, centrifuged, the clear phase aspirated and the remaining phase mixed with 1 ml 1x RBC lysis buffer and incubated for 3 min on ice. The reaction was stopped by adding PBS, cells were centrifuged, washed once in 1x RBC lysis buffer and resuspended in FACS buffer.

### MACS sorting

The CD11b^+^ samples for the microarray were generated using magnetic activated cell sorting (MACS). Following percoll gradient centrifugation, tumor and control cell pellets were resuspended in PBS, containing 0.5% FCS and 2 mM EDTA and labeled with anti-CD11b microbeads (Miltenyi Biotec, 130-093-634). The MACS isolation was carried out according to the manufacturer’s instructions and cells were subsequently used for RNA isolation.

### FACS sorting

Samples for qRT-PCR validation of target genes that were generated from GL261-bearing and corresponding naïve control mice were FACS sorted using CD11b, CD45, Ly6C, and Ly6G to distinguish between resident microglia (CD11b^+^/CD45^low^) and invading macrophages/monocytes (CD11b^+^/CD45^high^/Ly6G^-^/Ly6C^high^). Following percoll gradient centrifugation, cell pellets were resuspended in FACS buffer (containing 2% FCS) and stained with 2 μl of dye-coupled antibodies per 1*10^7^ cells. The staining was done with CD45-e450 (48-0451-82), Ly6G-PE (12-5931-82), Ly6C-PerCpCy5.5 (45-5932-82) and CD11b-APC (17-0112-82) (all eBioscience, San Diego, CA, USA) for 30 min at 4°C. Thereafter, the cells were washed and resuspended in 500 μl FACS buffer per 5*10^6^ cells for sorting at a BD FACSAria II (BD Bioscience). Compensation was calculated with single-stained beads (552844, BD Bioscience, Franklin Lakes, NJ, USA) and unstained cells.

Samples generated from RCAS-PDGFb tumors were isolated without antibody staining. Instead, tumor cells were reimplanted into *Cx3cr1* ^*GFP/wt*^ *Ccr2* ^*RFP/wt*^ mice which contain RFP^+^/GFP^low^ macrophages/monocytes and RFP^-^/GFP^+^ microglia. Accordingly, fluorescence-activated cell sorting was based on RFP and GFP positivity, in order to distinguish between resident microglia and invading macrophages/monocytes. RFP^+^/GFP^low^ peripheral macrophages/monocytes resemble the CD11b^+^/CD45^high^/Ly6G^-^/Ly6C^high^ population. The RFP^-^/GFP^+^ microglia resemble the CD11b^+^/CD45^low^ population.

### RNA Protocols

Total RNA from MACS or FACS-sorted cells was isolated using the RNeasy Plus Mini Kit (Qiagen, Hilden, Germany). On-column DNase 1 (Qiagen) digestion was performed and total RNA was eluted in RNase-free water. RNA yield was measured using a Nanodrop 1000 (Nanodrop, Wilmington, DE, USA) spectrophotometer and quality was assessed using an Agilent 2100 Bioanalyzer (Agilent, Santa Clara, CA, USA). Samples were stored at -80°C until further use. For qRT-PCR, first strand cDNA synthesis of RNA was done using the Superscript II (Invitrogen) reverse transcriptase according to the manufacturer’s instructions. For mRNA transcription oligo-dT primers (Invitrogen) were used. cDNA was stored at -20°C until further processing.

### Microarray

For the microarray MACS-isolated CD11b^+^ cells from GL261 gliomas (injected into 6–8-week-old C57BL/6 mice; 3 samples; one tumor sample per RNA sample), and age-matched naïve control mice (3 samples; three control brains were pooled per sample). We obtained yields of 500ng to 1 μg of total RNA of good quality (Agilent RNA Integrity Number, RIN = 8.2–10.0).

Sample preparation for microarray hybridization was carried out as described in the Ambion WT Expression Kit Protocol (Life Technologies, Carlsbad, CA, USA) and the Affymetrix WT Terminal Labeling and Hybridization User Manual (Affymetrix, Inc., Santa Clara, CA, USA). In brief, 300 ng of total RNA were used to generate double-stranded cDNA. 12 μg of subsequently synthesized cRNA was purified and reverse transcribed into sense-strand (ss) cDNA, whereat unnatural dUTP residues were incorporated. Purified ss cDNA was fragmented using a combination of uracil DNA glycosylase (UDG) and apurinic/apyrimidinic endonuclease 1 (APE 1) followed by a terminal labeling with biotin. 3,8 μg fragmented and labeled ss cDNA were hybridized to Affymetrix Mouse Gene 1.0 ST arrays for 16 h at 45°C in a rotating chamber. Hybridized arrays were washed and stained in an Affymetrix Fluidics Station FS450, and the fluorescent signals were measured with an Affymetrix GeneChip Scanner 3000 7G.

Sample processing was performed at an Affymetrix Service Provider and Core Facility, “KFB—Center of Excellence for Fluorescent Bioanalytics” (Regensburg, Germany; www.kfb-regensburg.de). Data sets are available at http://www.ebi.ac.uk/arrayexpress under the accession number E-MTAB-2660.

### Bioinformatic Analysis

Two additional data sets consisting of three samples each that were generated from CD11b^+^ MACS-isolated peritoneal myeloid cells were downloaded from http://www.ebi.ac.uk/arrayexpress [25]—E-MEXP-3623; [26]—E-GEOD-25585) and used as peripheral controls. Two independent approaches were used for bioinformatic data analysis. For the first method the Affymetrix Expression Console Software Version 1.0 was used to create summarized expression values (CHP-files) from the expression array feature intensities (CEL-files). The Robust Multichip Analysis (RMA) algorithm was applied [27]. Integrative analysis of genome-wide expression activities from DNA-microarray datasets was performed with the Gene Expression Dynamics Inspector (GEDI), a Matlab (Mathworks, Natick, MA) freeware program which uses self-organizing maps (SOMs) to translate high-dimensional data into a 2D mosaic [28]. Each tile of the mosaic represents an individual SOM cluster and is color-coded to represent high or low expression of the cluster's genes, thus identifying the underlying pattern.

For the second approach basal expression values (from CEL-files) were processed in R using Bioconductor package Affy [29]. The Affy-scale-value-expression-set-function was set to 500 and data was normalized with expresso-function, set to quantile normalization, and pmonly correcte.method and medianpolish summary method. Gene annotation was done with mogene10sttranscriptcluster [30], and only annotated probes were included in the analysis. Subsequently, collapseRows, was used to obtain a single representative probe for each gene, resulting in 13,943 genes taken into the analysis [31]. Signed network analysis was done with Weighted Gene Coexpression Network Analysis (WGCNA [32]). An adjacency network was made using a softpower (β) value of 20, based upon the scale free criteria, and low connected genes were filtered out of the analysis, resulting in 10,875 genes. Dendrogram formation and module determination was done by average linkage clustering and an arbitrary cut-off, determined by WGCNA. From each module, the Module Eigengene (ME) was calculated, which is the first principal component and functions as a representative of the expression profile of the module. Next, as a measure for intramodular connectivity, the ME was correlated to the expression profile of all intramodular genes, resulting in a kME-table, to determine which genes are most important, e.g. hub-genes. These module Membership values were multiple testing corrected using R package Stats. Genes significantly associated to these modules (FDR p value <0.01) were used for further analysis.

Heat maps were generated using heatmap.2 function of Bioconductor Package Gplots [33]. Functional annotation and transcription factor binding site enrichment analysis of the modules was done using Webgestalt [34,35]. The WGCNA UserlistEnrichment method was used to determine the significance of the overlap of several gene lists with the induced gene lists using a Hypergeometric test [31].

For the Gene Set Enrichment Analysis (GSEA) the 438 upregulated genes from our GAMs dataset were taken to form a gene set, and it was tested if this gene set is overrepresented in the upregulated or downregulated genes in the M1 or M2a,b,c macrophage data sets in comparison to M0. For each of M1, M2a, M2b, and M2c polarization, in total 4 runs of GSEA were executed. Taking M1 as an example, we fed the gene expression values of M1 and M0, each with 3 replicates, to the GSEA software; signal-to-noise ratios of each gene were calculated to rank the genes in a descending manner. An enrichment score (ES) for the gene set of 438 genes was then computed based on the gene ranking, which corresponds to the magnitude of the overrepresentation. Thereafter, the significance level of the ES was evaluated by a gene-set permutation test with 1000 permutations.

### qRT-PCR

Quantitative RT-PCR reactions were performed using the SYBR Select Mastermix (Applied Biosystems, Foster City, CA, USA) according to the manufacturer’s instructions on a 7500 Fast Real-Time PCR System (Applied Biosystems).

Primers for target genes were designed to recognize all validated mRNA splice variants of the gene, relying on the RefSeq sequences of the UCSC genome browser (http://genome.ucsc.edu/). The primer sequences were generated using the Primer-Blast tool (http://www.ncbi.nlm.nih.gov/tools/primer-blast/). Amplification of unintended targets was excluded by BLAST search (http://blast.ncbi.nlm.nih.gov/Blast.cgi). Calculation of unfavorable secondary structures and primer-dimer amplification was done with the IDT oligo analyzer (http://eu.idtdna.com/analyzer/applications/oligoanalyzer/). For absolute quantification of target genes, nested primers flanking the original primers were designed. Nested amplicons were purified from agarose gels, and serial dilutions were used to generate standard curves for each gene. Primer sequences can be found in S1 Table.

### Survival outcome analysis

Cbioportal (http://www.cbioportal.org/public-portal/) was used to access patient survival information and tumor gene expression data of GBM patients (Study: Glioblastoma multiforme, TCGA Provisional, mRNA Expression z-Scores (microarray), accessed in August 2014) [36,37]. Subtype information was retrieved from [38]. The standard deviation of the gene expression values of all patients was calculated. Patients with a gene expression lower than the negative standard deviation were clustered into the low expression group, whereas patients with a gene expression higher than the positive standard deviation were clustered into the high expression group. For subtype analysis the patients were clustered according to the standard deviation of gene expression within that subtype. Survival plots were generated and Kaplan-Meier curves were produced and statistical significances between high and low expression groups were calculated with the Log Rank (Mantel-Cox) test using GraphPad Prism 5. To test for a proportional risk increase with each unit of gene expression, we fitted a Cox proportional hazards regression model to our data including a test for the proportional hazards assumption [39]. Analysis was done using the statistical script language R [40].

## Results

### Gene expression profile of glioma-associated microglia/macrophages (GAMs)

To identify glioma-regulated transcripts in glioma-associated microglia/macrophages, we isolated microglia/macrophages from GL261 glioma-bearing brains using MACS sorting with anti-CD11b antibodies. As controls we isolated microglia from age-matched healthy mouse brain and performed an Affymetrix Genechip Mouse Gene 1.0ST microarray on these samples. Additionally, we also used two data sets that were generated from CD11b^+^ MACS-isolated peritoneal myeloid cells as peripheral controls. These data sets were downloaded from http://www.ebi.ac.uk/arrayexpress [25]—E-MEXP-3623; [26]—E-GEOD-25585). The two additional data sets consisted of three samples each, generated from either CD11b^+^ MACS-sorted [26] or CD11b^+^MHC^-^II^hi^B220^-^Gr1^-^ flow-sorted cells [25]. When comparing the GAMs data set to the three naïve control data sets, 783 genes were significantly upregulated at least 2-fold and 198 genes downregulated at least 2-fold (S2 Table).

**Fig 1:**
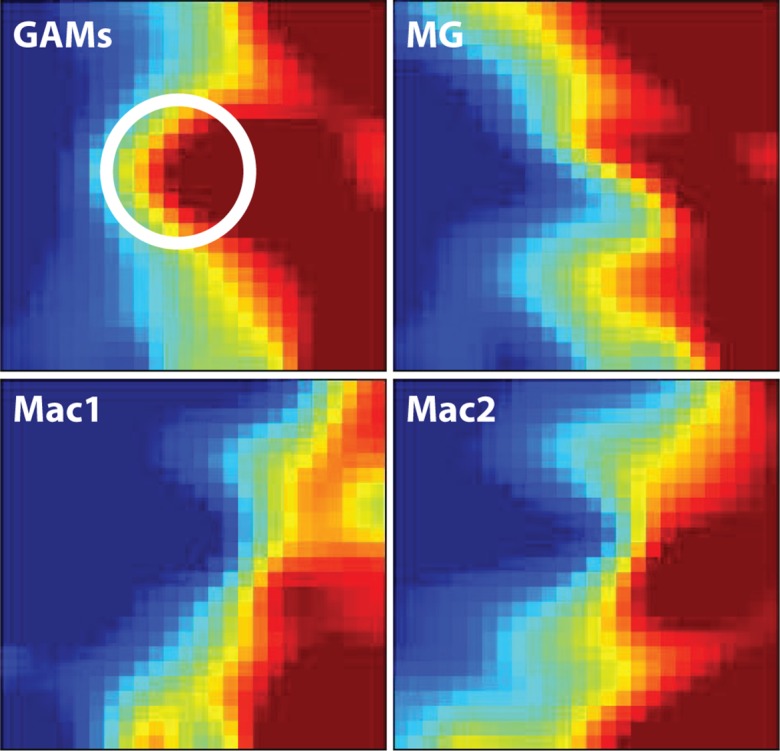
Graphical representation of gene expression patterns in the four data sets. The four GEDI maps show that the gene expression patterns of GAMs and naive microglia are more similar to each other than both macrophage datasets (blue colour indicating low and red colour high mRNA expression levels). The white circle highlights a central cluster of highly expressed genes in GAMs that is different from naive microglia. GAMs: glioma-associated microglia/macrophage microarray data; MG: naive microglia microarray data; Mac1: external data set from Keller, Mazuch et al. 2009; Mac2: external data set from Young, Eksmond et al. 2012.

We first applied the Gene Expression Dynamics Inspector (GEDI) on these datasets to visualize the global patterns of gene expression in GAMs versus naive microglia and macrophages. GEDI uses self-organizing maps to capture genome-wide transcriptome activity via ‘gestalt’ recognition [28]. GEDI facilitates the identification of genome-wide patterns with each mosaic tile in the map representing a gene cluster that is expressed at similar levels. The four GEDI maps, with blue colour indicating low and red colour high mRNA expression levels, show that the gene expression patterns of GAMs and naive microglia are more similar to each other than both macrophage datasets. Closer inspection of both microglia GEDI patterns then revealed a central cluster of highly expressed genes in GAMs that is different from naive microglia (white circle) (Fig. 1).

### Weighted Gene Coexpression Network Analysis of the data sets

**Fig 2:**
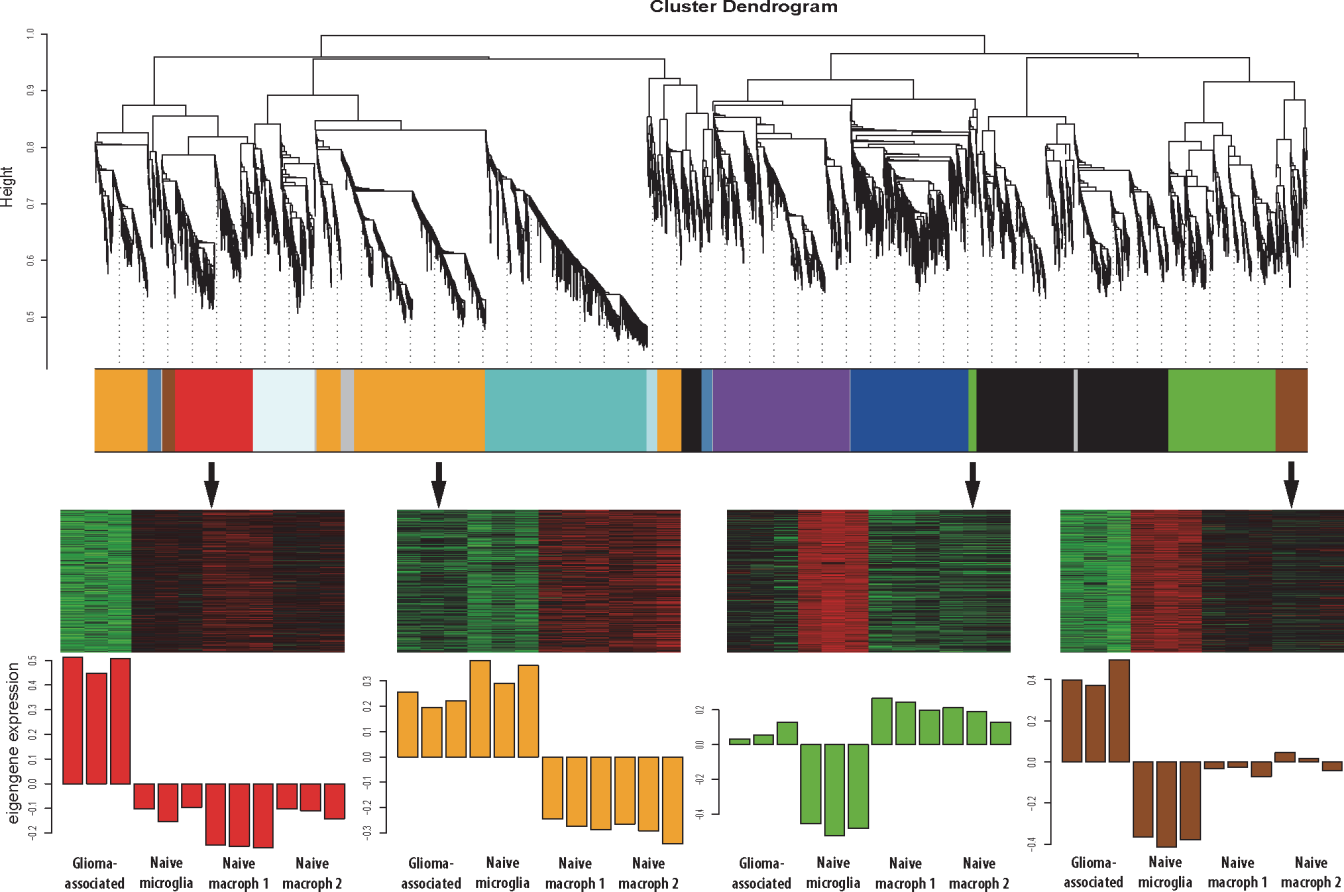
WGCNA gene clustering reveals glioma-regulated gene modules. Each color represents a different module and each module contains genes with similar expression patterns over all four sample sets. The glioma-regulated modules (labeled as red and brown) were further analyzed, as they contained genes that were upregulated in the glioma-associated set when compared to all three control sets. A third module (labeled as green module) contained genes that were highly expressed in GAMs when compared to naïve microglia, but were already highly expressed in peripheral macrophages. The module that is depicted as orange contains genes that were highly expressed in GAMs, and also highly expressed in naïve microglia, however expressed at lower levels in peripheral macrophages. All other clusters contained genes that did not seem to be glioma-regulated.

Using Weighted Gene Coexpression Network Analysis (WGCNA), we clustered genes into different modules, according to their co-expression pattern (Fig. 2). Within each module genes were ranked according to how closely they correlated to the Eigengene expression of this module (termed Module Membership). The Module Eigengene is the first principal component, representing the gene expression patterns of all genes clustered into this module [41]. Two modules contained genes that were upregulated in glioma-associated samples compared to the three control data set (labeled red and brown in Fig. 2). Table 1 shows the genes with the strongest upregulation in GAMs and the modules they were clustered into. S3 Table lists all 10,875 genes and the corresponding modules they were clustered into.

**Table 1:** The 25 highest-upregulated genes in the glioma-associated data set compared to all three control sets.

			Log2 Expression				
Gene Symbol	Module	P Value	GAMs	Naïve Microglia	Control Mϕ1	Control Mϕ2	Fold Change
Gpnmb	red	2.06E-07	12.63	7.26	7.60	7.51	36.02
Cxcl9	red	1.69E-08	12.83	7.63	7.14	8.67	32.27
Spp1	red	7.59E-06	12.31	7.92	7.84	6.88	27.24
Ly6i	red	6.84E-09	11.55	6.94	6.79	7.07	24.48
Gjc3	red	1.41E-08	11.62	7.74	6.97	7.13	20.32
Il1r2	red	1.86E-06	11.64	8.22	7.03	6.99	18.70
Cd72	red	1.63E-06	12.13	8.81	7.63	7.42	18.04
Ms4a4c	red	2.36E-07	12.11	7.72	8.00	8.15	17.76
Mmp13	red	2.34E-07	11.08	7.11	6.77	6.91	17.73
Serpina3n	red	3.08E-08	11.71	8.18	7.17	7.39	17.45
Ptgs2	orange	0.001883	12.61	11.09	7.167	7.23	17.28
Gpr171	red	1.02E-07	11.23	7.20	7.21	7.24	16.12
Igfbp7	orange	0.00065	12.52	10.53	7.59	7.49	15.83
Il1b	orange	0.001877	12.71	11.25	7.42	7.58	15.54
Ccl5	red	1.58E-10	12.46	9.02	8.00	8.64	15.03
Adamdec1	red	2.47E-07	10.69	6.75	6.81	6.84	14.78
Il1rn	red	1.99E-10	11.63	8.03	7.46	7.77	14.76
Ifi44	red	3.59E-07	11.42	7.53	7.27	7.95	14.32
Spon1	red	4.10E-07	11.77	8.72	7.52	7.55	14.30
Acp5	brown	0.000145	12.38	7.74	9.24	8.88	13.58
Moxd1	red	4.40E-08	10.83	7.21	7.15	7.11	12.77
Trem1	red	7.96E-07	11.43	8.10	7.65	7.57	12.62
Gm6377	red	2.25E-06	11.14	8.45	7.05	7.02	12.40
Vcam1	orange	0.000315	11.76	9.95	7.19	7.36	12.12
Dnase1l3	brown	0.000265	10.98	6.91	7.68	7.66	11.84

In addition, the gene clustering revealed genes that were specific for either microglia or macrophages. The orange module contains genes that were highly expressed in GAMs and already showed high expression in naïve microglia, but low expression in naïve macrophages (Fig. 2). Genes that showed high expression in GAMs and naïve macrophages, but low expression in naïve microglia were clustered into the green module (Fig. 2). Two studies used RNA sequencing and microarray analysis to determine microglia and macrophage-specific genes [42,43]. Genes that were found to be microglia-specific in these reports predominantly clustered into our orange module, whereas macrophage-specific genes are enriched in our green module, validating our findings.

We used Webgestalt to perform gene ontology (GO) enrichment analysis on all >2 fold upregulated genes that clustered into the glioma-regulated (red and brown) modules (438 genes in total) to identify overrepresented GO terms (Table 2). Several GO terms were overrepresented in the GAMs data set that can be grouped into three main groups: regulation of immune response/activation, programmed cell death, and response to other organism/to virus. All genes included in the overrepresented GO terms are listed in S4 Table. Next, we used Webgestalt to identify transcription factor binding sites that are enriched in the same set of genes (Table 3). This analysis revealed that enriched binding sites included known sites for IRF1, IRF2, IRF7, IRF8, IRF9/STAT1/STAT2, STAT5A, STAT5B, and NFAT. Both *Stat1* and *Stat2* clustered into the glioma-regulated brown module and were around 2.5 fold upregulated. In contrast, *Stat5a* and *Stat5b* were not significantly regulated at the transcriptional level. *Irf7* and *Irf9* were 6.9-fold and 1.4-fold upregulated and clustered into the brown and red module, respectively.

**Table 2:** We used Webgestalt to identify overrepresented GO terms in >2 fold upregulated genes in the glioma-regulated (red and brown) modules.

Biological process	Number of genes	Adjacent p value
		
Immune system process	102 genes	adjP = 1.21e-30
Immune response	72 genes	adjP = 9.65e-30
Defense response	68 genes	adjP = 6.25e-23
Innate immune response	44 genes	adjP = 6.25e-23
Response to biotic stimulus	53 genes	adjP = 3.70e-22
Response to other organism	51 genes	adjP = 5.91e-22
Immune effector process	45 genes	adjP = 1.47e-18
Response to stress	106 genes	adjP = 7.91e-18
Response to cytokine stimulus	40 genes	adjP = 1.23e-17
Regulation of immune system process	57 genes	adjP = 1.40e-17
Multi-organism process	55 genes	adjP = 2.53e-16
Response to virus	25 genes	adjP = 1.19e-13
Leucocyte activation	39 genes	adjP = 2.52e-10
Cell activation	41 genes	adjP = 7.62e-10
Programmed cell death	67 genes	adjP = 1.24e-09
Cell death	69 genes	adjP = 2.22e-09

**Table 3:** We used Webgestalt to identify enriched transcription factor binding sites in >2 fold upregulated genes in the glioma-regulated (red and brown) modules.

Enriched transcription factor targets	Genes	Statistics
mmu_STTTCRNTTT_V$IRF_Q6	Bst2, Ddr2, Dhx58, Dtx3l, Epsti1, Hgf, Ifi44, Ifit2, Ifit3, Il18bp, Isg15, Lgals3bp, Nampt, Tap1, Tnfsf13b, Usp18, Xaf1, Zbp1	adjP = 2.16e-05
mmu_V$ISRE_01	Ammecr1, Bst2, Cxcr4, Dhx58, Dtx3l, Epsti1, Gpr65, Ifi44, Ifih1, Ifit2, Ifit3, Isg15, Kynu, Met, Mmp25, Pgk1, Tnfsf13b, Usp18, Xaf1, Zbp1	adjP = 2.84e-05
mmu_V$ICSBP_Q6	Adam8, Bst2, Cxcr4, Dtx3l, Emp1, Ifi44, Ifih1, Ifit3, Il18bp, Isg15, Kynu, Parp12, Tap1, Tfec, Tnfsf13b, Usp18, Zbp1, Zmynd15	adjP = 0.0004
mmu_V$IRF7_01	Bst2, Cxcr4, Dll4, Dtx3l, Epsti1, Ifit2, Il18bp, Isg15, Lgals3bp, Nr4a2, Nr4a3, Parp12, Pdgfc, Tap1, Usp18, Xaf1, Zmynd15	adjP = 0.0012
mmu_V$IRF_Q6	Bst2, Cd80, Cxcr4, Dhx58, Dnase1l3, Dtx3l, Fcgr2b, Ifi44, Ifit2, Il18bp, Isg15, Kynu, Parp12, Tnfsf13b, Ube2l6, Zbp1	adjP = 0.0026
mmu_V$IRF1_01	Bst2, Ccl5, Dnase1l3, Dtx3l, Isg15, Kynu, Neto1, Pdgfc, Slamf8, Tap1, Tfec, Tgfb3, Tnfsf13b, Usp18, Xaf1	adjP = 0.0083
mmu_TTCYNRGAA_V$STAT5B_01	Ccl5, Cish, Crem, Dll4, Gzmb, Il18bp, Nfil3, Nkg7, Nr4a3, Pcolce, Plagl1, Plscr1, Serping1, Socs2, Stc1, Tnfrsf9, Trim25	adjP = 0.0213
mmu_TGGAAA_V$NFAT_Q4_01	Adam9, Adamtsl4, Adm, Aig1, Arrdc4, Bhlhe40, Ccl5, Cd72, Cdkn1a, Chl1, Cish, Creb5, Crem, Ctgf, Ctla4, Dab2, Ddr2, Dll4, Emp1, Erbb3, Gsn, Has2, Hgf, Hif1a, Htr7, Htra4, Ifng, Igfbp3, Il1rn, Impa2, Inhba, Irs2, Isg15, Kynu, Lgals1, Mcam, Mdfic, Mmp14, N4bp1, Nfil3, Nr4a2, Nr4a3, Pde4b, Pdk3, Pgam1, Plod2, Prr11, Prrx1, Sema6a, Socs2, Spp1, Stc1, Tgfb3, Tmem97, Tnfsf10, Trim25, Vegfa	adjP = 0.0221
mmu_V$STAT5B_01	Ccl5, Cish, Crem, Dll4, Nfil3, Nr4a2, Nr4a3, Pcolce, Plagl1, Plscr1, Serping1, Socs2, Stc1, Trim25	adjP = 0.0221
mmu_V$STAT5A_01	Ccl5, Cish, Crem, Dll4, Nfil3, Nr4a2, Nr4a3, Pcolce, Plagl1, Plscr1, Serping1, Socs2, Stc1, Tnfrsf9	adjP = 0.0249
mmu_TGANTCA_V$AP1_C	Adm, Aig1, Ass1, Atp6v0d2, Ccr7, Cd109, Cdkn1a, Creb5, Cst7, Emp1, Furin, Gpnmb, Gpr141, Gzmb, Hspb6, Il10, Il1rn, Isg20, Itgax, Krt8, Lgals1, Mmp12, Mmp13, Mmp19, N4bp1, Osmr, Plp2, Procr, Prrg4, Stat1, Stc1, Tm4sf19, Tnfrsf9, Trim25, Vat1, Vdr, Vegfa	adjP = 0.0460
mmu_V$STAT_01	Cish, Crem, Gzmb, Il18bp, Nfil3, Nkg7, Nr4a3, Pcolce, Plscr1, Runx3, Serping1, Socs2, Trim25	adjP = 0.0460
mmu_V$IRF2_01	Bst2, Dnase1l3, Dtx3l, Pdgfc, Tap1, Tgfb3, Tnfsf13b, Usp18, Xaf1	adjP = 0.0460

### GAMs transcriptome only partially resembles an M1 or M2 polarization

To further analyze the phenotype of GAMs, we compared our glioma-associated data set with typical markers for an M1 and M2 macrophage phenotype (Table 4). Furthermore, we compared our data set with data sets of macrophages that were polarized *in vitro* into an M1 or M2a,b,c phenotype. We used data sets (Data set: E-GEOD-32690; [44]) that contained data of macrophages stimulated *in vitro* for 24 h with LPS and IFN-γ (M1 polarization), IL4 (M2a polarization), IFN-γ and complexed Ig (M2b polarization), and Dexamethasone (M2c polarization) – in comparison to unstimulated M0 macrophages.

**Table 4:** Expression of known M1, M2, and TAM marker genes in our GAMs data set.

	M1 markers			M2 markers			Markers tumor-associated Mφ		
	Genes	Log2 Expression	Regulation	Genes	Log2 Expression	Regulation	Genes	Log2 Expression	Regulation
Receptors and intracellular	Stat1	11.56	2.27	Tgm2	12.28	6.94	Cd204	11.87	13.55
	Tlr2	11.29	2.51	Cd206	12.15	1.54	Stat3	11.67	1.42
	Cd80	11.20	2.38	Cd204	11.87	13.55	Vegfr2	9.34	2.13
	Cd86	11.13	2.03	Il1rII	11.63	1.72	Met	8.08	2.60
	Tlr4	9.72	-1.33	Tlr8	11.00	3.21	Egfr	7.20	-1.52
	Il1rI	7.67	1.49	Tlr1	10.72	2.00	Cd163	7.10	-4.40
				Cd163	7.10	-4.40			
Secreted facrots and cytoskines	Il1b	12.71	15.54	Il1rn	11.63	14.76	Arg1	11.48	3.91
	Tnfa	11.03	2.89	Tgfb3	10.28	7.85	Mmp14	11.36	9.91
	Nos2	10.14	7.24	Il10	8.96	3.76	Vegfa	11.34	8.35
	Il18	9.33	1.71	Il6	8.32	1.90	Mmp13	11.08	17.73
	Ifng	8.56	2.57				Tnfa	11.03	2.89
	Il15	8.47	-1.36				Ctgf	10.53	5.35
	Il23a	7.28	1.41				Arg2	10.43	11.37
	Indol1	6.93	1.02				Mmp2	10.43	4.07
	Il12a	6.61	1.18				Tgfb3	10.28	7.85
							Hgf	10.12	4.13
							Il10	8.96	3.76
							Mmp9	8.08	-2.22
							Il12a	6.61	1.18
Chemokines	Ccl3	13.21	7.93	Ccl17	8.84	2.47	Cxcl10	12.83	10.60
	Cxcl9	12.82	32.27	Ccl24	8.05	-5.04	Ccl5	12.46	15.03
	Ccl5	12.46	15.03	Ccl22	7.98	1.61	Cxcl16	12.18	5.94
	Ccl2	11.15	4.86	Ccl1	6.86	1.22	Ccl17	8.84	2.47
	Ccl9	11.03	-1.47				Ccl22	7.98	1.61
	Ccl4	10.43	2.68				Cxcl12	7.01	-1.77
	Ccl8	10.25	10.52				Ccl18	6.20	1.02
	Ccl11	8.54	2.00						

Marker genes were taken from literature reviews [10,17,65].

**Fig 3:**
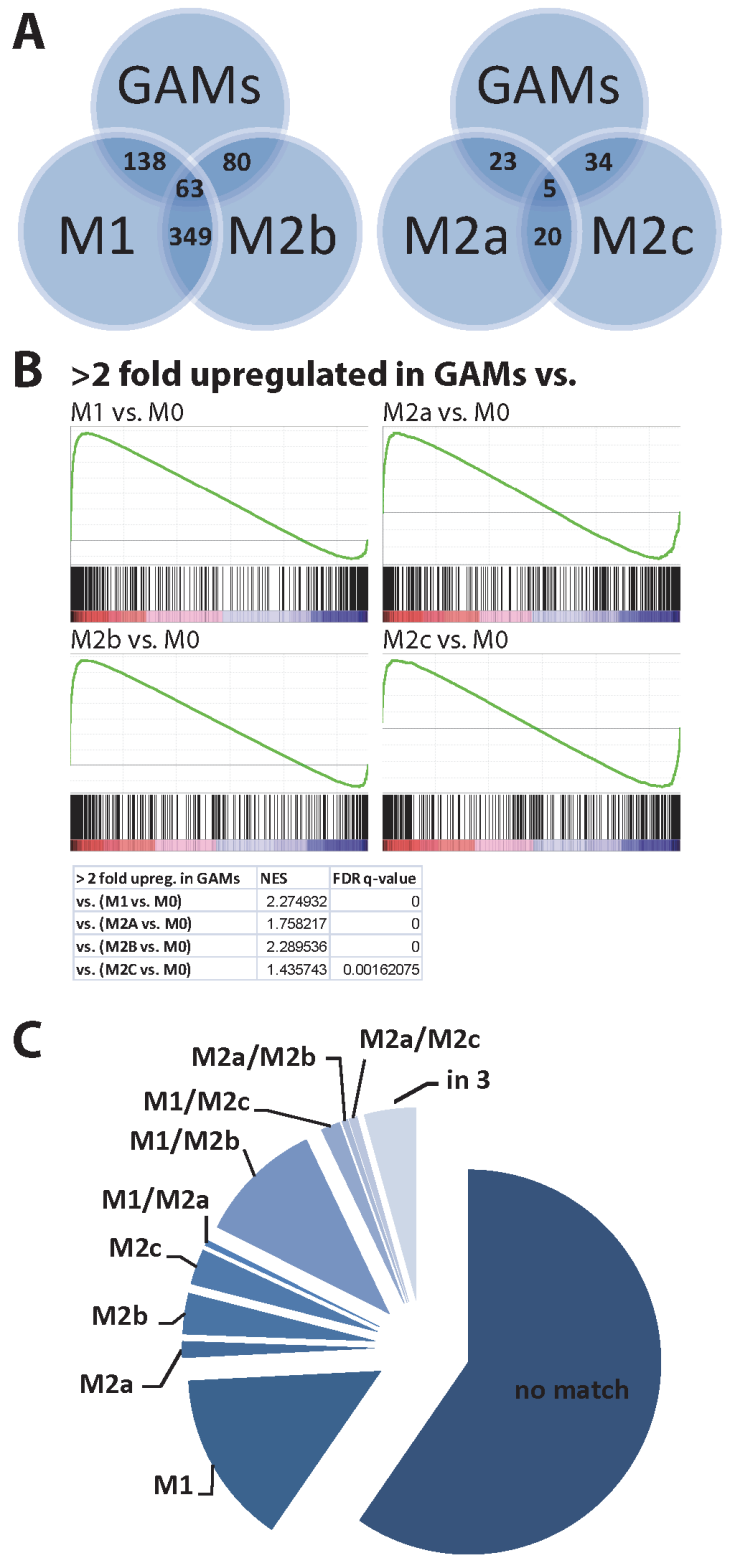
Comparison of GAMs with data sets of M1, M2a,b,c-stimulated macrophages. We retrieved data sets from http://www.ebi.ac.uk/arrayexpress (Data set: E-GEOD-32690; [44]), containing data of macrophages that were stimulated for 24 h *in vitro* into different polarization states (M0 (unstimulated), M1 (IFNγ + LPS), M2a (IL4), M2b (IFNγ + complexed Ig), and M2c (Dexamethasone)) and compared which genes in these data sets significantly overlapped with glioma-regulated genes in our GAMs data set. A: A graphical representation of the overlap of upregulated genes in GAMs and the four macrophage data sets. The GAMs gene expression profile shows the greatest number of overlaps with M1, and M2b polarized macrophages. B: Using Gene Set Enrichment Analysis (GSEA) we found that the 438 upregulated genes in GAMs are significantly enriched in the upregulated genes of all four macrophage polarization sets. C: Only a minority of genes upregulated in GAMs were also induced in the M1 to M2c phenotype, 59.5% of the genes that were upregulated in GAMs were not regulated in any of the four macrophage phenotypes.

For this analysis we considered genes in our GAMs dataset that were glioma-regulated (genes that clustered into the red and brown module) and were >2-fold upregulated and compared them with genes that were >2-fold up- or downregulated in the four M1/M2a,b,c data sets (in comparison to the M0 state). GAMs: 438 upregulated genes; M1: 1243 genes up-, 1704 genes downregulated; M2a: 227 genes up-, 322 genes downregulated; M2b: 501 genes up-, 483 genes downregulated; M2c: 381 genes up-, 358 genes downregulated. We found that all gene sets are significantly enriched in our GAM profile, but the M1 and M2b subsets were most significantly affected: M1 (138 out of 438 upregulated genes in GAMs were also upregulated in M1 macrophages, 31.5%, Bonferroni p value: 2.01e-60), M2b-polarized macrophages (80 out of 438 upregulated genes in GAMs were also upregulated in M2b macrophages, 18.3%, p value: 5.29e-43). The enrichment of M2c- (34 out of 381 genes, 7.8%, p value: 1.62e-11) and M2a (23 out of 438 genes, 5.3%, p value: 6.88e-09) was less strong (Fig. 3A). Table 5 lists all genes that are significantly enriched in our GAMs data set and are specific for either M1, M2a, M2b, or M2c polarization. Using Gene Set Enrichment Analysis (GSEA) we found that the 438 upregulated genes in GAMs are significantly enriched in the upregulated genes of all four macrophage polarization sets (Fig. 3B). Furthermore we tested the enrichment of the >2 fold up- and downregulated M1, M2a,b,c genes in the entire GAMs data set that resulted from the WGCNA analysis (10,875 genes) (S1 Fig.). Six out of eight sets were significantly enriched in the up- and downregulated genes of the GAMs data set. However, 59.6% of the genes that were upregulated in GAMs (261 out of 438 genes) were not upregulated in any of the four macrophage phenotypes (Fig. 3C). This indicates that the GAMs phenotype shows only partial overlap with the classical M1 or M2 macrophage phenotype.

**Table 5:** M1 and M2a,b,c-specific genes that are >2-fold upregulated in our GAMs data set.

	Genes
M1-specific	1500012F01Rik, 1600014C10Rik, Ahr, Arrdc4, BC023105, Best1, Bst2, C2, Car13, Ccr7, Cd200, Cd52, Clec4n, Clic4, Cpd, Crem, D16Ertd472e, Ell2, Epsti1, Ero1l, Fmnl2, Gpr132, Gpr31c, Hmox1, Htra4, Ifi204, Ifih1, Il1rn, Inhba, Irf7, Isg20, Itga5, Kynu, Malt1, Mefv, Met, Mmp14, Mmp25, Mxd1, Nos2, Nr1h3, Nr4a2, Oas3, Oasl2, Parp12, Plagl1, Ppa1, Procr, Pvr, Rab11fip1, Rasgrp1, Rnf213, Runx3, Slamf7, Slfn5, Srxn1, Stat2, Timp1, Tiparp, Tnfaip2, Treml4, Trim25, Ube2l6, Zmynd15
M2a-specific	Atp6v0d2, Clec7a, Itgax, Mmp13, Tnfrsf26, Vwf
M2b-specific	AI504432, Casp12, Gpr171, Ifitm1, Il12rb2, Impa2, Itga1, Pdgfc, Plac8, Rspo1, Tgfb3, Tgfbi, Tmem171, Tnfsf13b, Vdr
M2c-specific	Adamtsl4, Amica1, Arhgap19, Ctla2a, Cxcr4, Cyp4f18, Fbxo32, Gpr35, Gpx3, Il1r2, Ldlrad3, Mmp19, Wbp5

### Validation of M1 and M2a,b,c marker gene expression in GAMs

In addition we selected five genes that were upregulated in GAMs, clustered into the glioma-regulated (red and brown) modules, and were specifically upregulated in either M1, M2a, M2b, or M2c-polarized macrophages and validated the expression of these genes by qRT-PCR in FACS-sorted samples. We selected *Il1rn, Isg20* (both specific for M1-polarized macrophages), *Clec7a* (M2a), *Tgfbi* (M2b), and *Cxcr4* (M2c).

**Fig 4:**
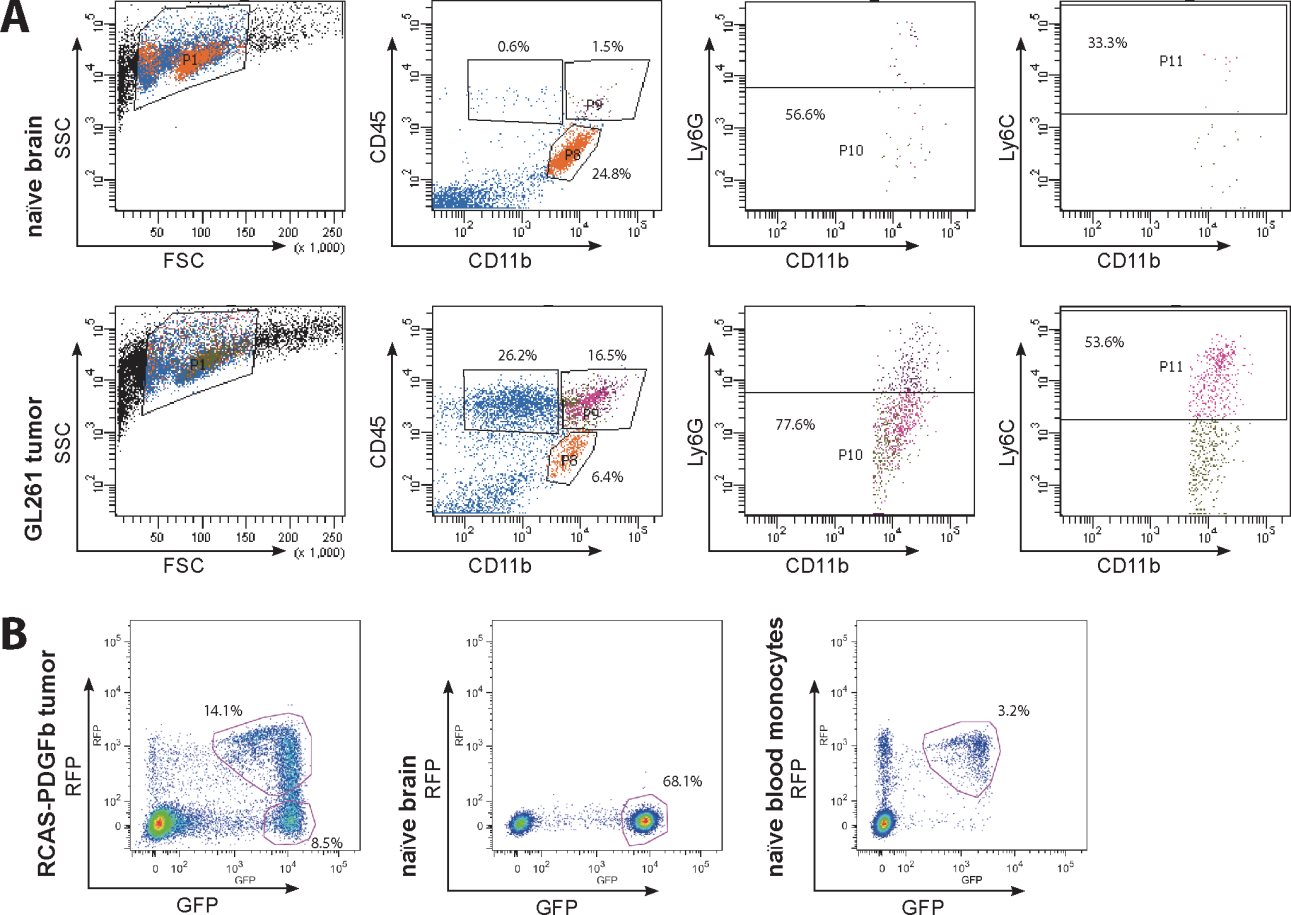
Flow cytometry isolation strategies for generation of mouse qPCR samples. A) Plots depicting the strategy for isolation of microglia and macrophages from naïve brains and GL261 glioma-bearing brains using antibody staining for CD11b, CD45, Ly6C, and Ly6G. CD45 staining was used to distinguish between CD11b^+^/CD45^low^ resident microglia (gate P9) and CD11b^+^/CD45^high^/Ly6G^-^/Ly6C^high^ invading macrophages/monocytes (gates P8, P10, and P11), which were mostly absent in naïve brain samples. B) GAMs from RCAS-PDGFb tumors were isolated relying on an antibody-independent approach. Primary tumors from *Ntv-a*/*Ink4a-Arf^-/-^* mice were reimplanted into *Cx3cr1* ^*GFP/wt*^ *Ccr2* ^*RFP/wt*^ mice and GAMS were sorted according to GFP and RFP expression. In naïve *Cx3cr1* ^*GFP/wt*^ *Ccr2* ^*RFP/wt*^ mice only GFP^+^ cells were present in the brain. In tumor-bearing mice, resident microglia were GFP^+^, whereas invading macrophages/monocytes were RFP^+^/GFP^low^ or GFP^+^/RFP^+^. RFP^+^/GFP^low^ naïve blood monocytes were used as peripheral controls.

We FACS-isolated GAMs from GL261 tumors and RCAS-PDGFb tumors and measured the expression by qRT-PCR in comparison to microglia and peripheral macrophages isolated from control animals. For GAMs isolated from the GL261 mouse model, we distinguished between resident microglia (CD11b^+^/CD45^low^) and invading macrophages/monocytes (CD11b^+^/CD45^high^/Ly6G^-^/Ly6C^high^) by FACS sorting (Fig. 4A; [45]). In addition to microglia derived from naïve animals we also used spleen-derived macrophages/monocytes as additional controls.

As a further validation we used the RCAS-PDGFb tumor model, which produces pro-neural high-grade glioma, as a second glioma model system [22]. For this tumor model primary RCAS-PDGFb tumors were re-transplanted into *Cx3cr1* ^*GFP/wt*^ *Ccr2* ^*RFP/wt*^ mice, which allowed us to FACS-isolate RFP^-^/GFP^+^ microglia and RFP^+^/GFP^low^ macrophages/monocytes from tumors, as well as RFP^-^/GFP^+^ microglia from control brains and RFP^+^/GFP^low^ monocytes from peripheral blood without further antibody staining (Fig. 4B; [23]).

**Fig 5:**
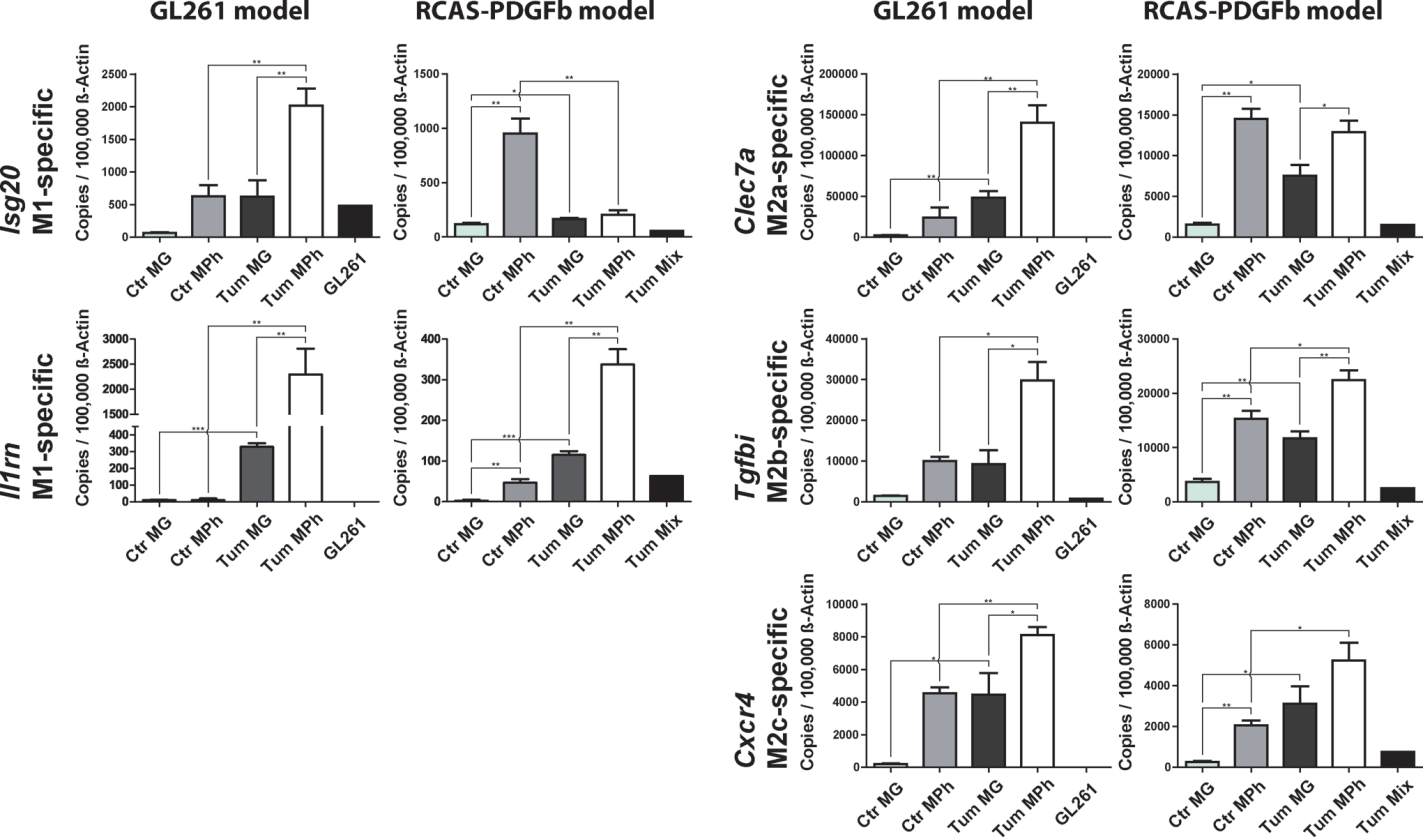
qPCR validation of selected M1 and M2a,b,c-specific genes in murine GAMs. We selected 5 genes that were upregulated in GAMs and specific for either M1 (*Il1rn* and *Isg20*), M2a (*Clec7a*), M2b (*Tgfbi*), or M2c polarization (*Cxcr4*) and investigated the expression of these using qRT-PCR. For this we isolated GAMs from GL261 and RCAS-PDGFb tumors using flow cytometry, in order to distinguish between resident microglia and invading macrophages/monocytes and used microglia, and spleen-derived macrophages/monocytes from naïve mice as controls. CTR MG: naïve microglia, CTR Mph: naïve monocytes, Tum MG: glioma-associated microglia, Tum Mph: glioma-associated macrophages/monocytes, GL261: cultured GL261 cells, Tum Mix: cultured RCAS-PDGFb tumor cells. Bar graphs illustrate the absolute number of transcripts normalized to 100,000 transcripts of *Actb* (n = 4). Analysis was done by students t test. Error bars indicate the Standard Error of Mean (SEM). *, p<0.05; **, p<0.01; ***, p<0.001

In the GL261 model a significant higher expression of all selected genes could be confirmed in glioma-associated macrophages/monocytes and for *Il1rn, Clec7a*, and *Cxcr4* in glioma-associated microglia, compared to the respective control cells (Fig. 5).

In the RCAS model we saw a significant higher expression of all selected genes when comparing glioma-associated microglia to naïve microglia. When comparing glioma-associated macrophages/monocytes to naïve monocytes, only the expression of *Il1rn, Tgfbi*, and *Cxcr4* was significantly higher. For *Isg20* we detected a lower expression in glioma-associated macrophages/monocytes when compared to naïve monocytes. Furthermore, the expression of *Clec7a* was unchanged in glioma-associated macrophages/monocytes when compared to naïve monocytes. All investigated genes were expressed at higher levels in naïve monocytes when compared to naïve microglia. In part the expression was as high as in glioma-associated microglia. The difference of gene expression between the RCAS and the GL261 model might be partially caused by the different isolation procedures. We used *GFP/RFP* expression in *Cx3cr1* ^*GFP/wt*^ *Ccr2* ^*RFP/wt*^ mice for the RCAS model, as compared to antibody staining for CD11b/CD45/Ly6G/Ly6C in the GL261 model.

Furthermore, the expression of the selected genes was lower in the RCAS model when compared to the GL261 model – this was most notable for *Isg20, Il1rn*, and *Clec7a*. This might represent differences in the tumor biology of both models, but might also be owed to the different growth pattern and kinetics of the tumors. Mice injected with GL261 tumors were sacrificed 20 days post-injection, whereas mice implanted with RCAS tumors were sacrificed 4–5 weeks post-operation. Thus, GAMs in RCAS tumors were exposed to the tumor environment over longer times and the transcriptional program might have changed over the duration of the stimulus.

**Fig 6:**
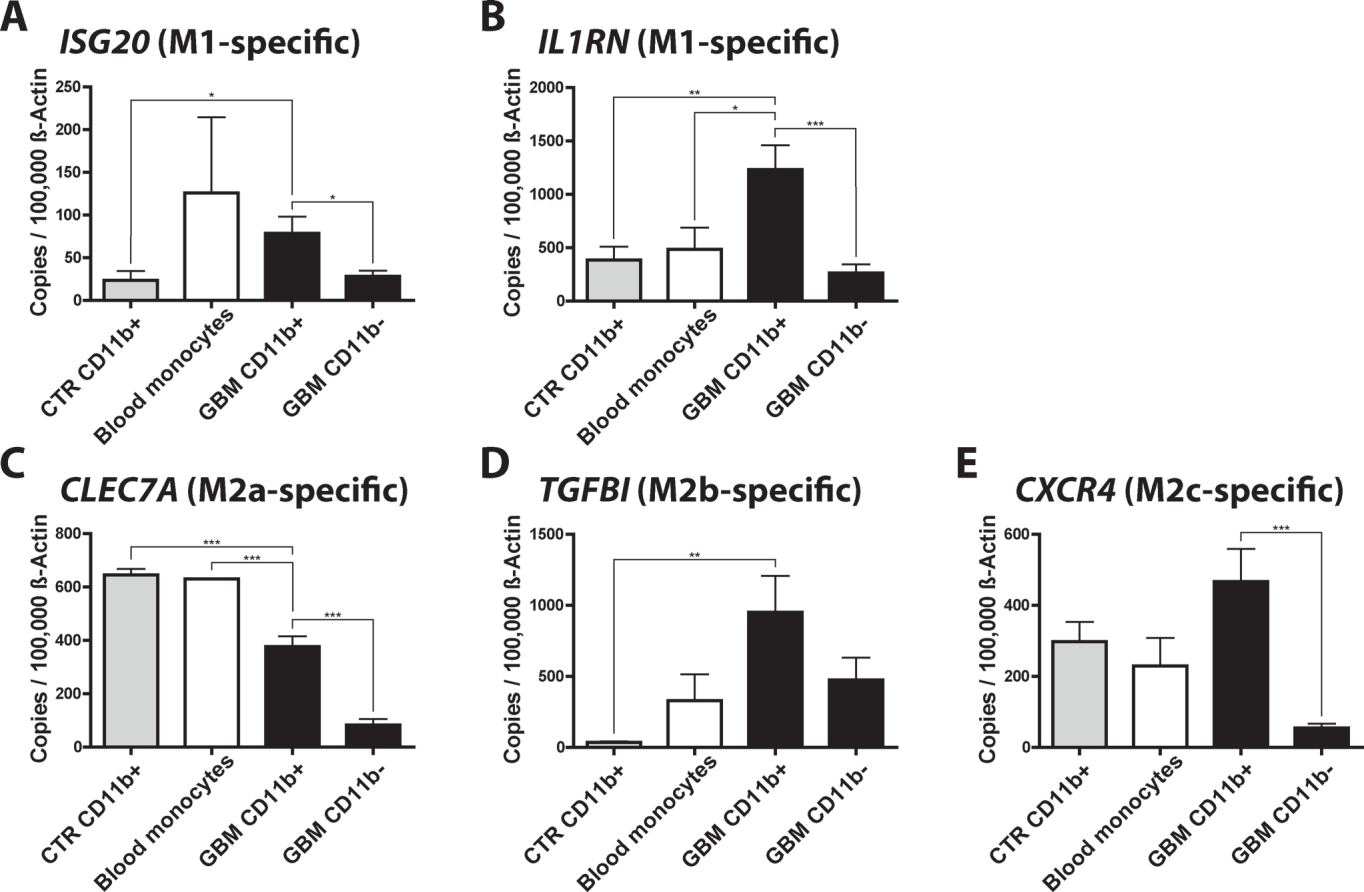
qPCR validation of selected M1 and M2a,b,c-specific genes in human GAMs. We determined the expression of the M1 (*IL1RN* and *ISG20*), M2a (*CLEC7A*), M2b (*TGFBI*), and M2c-specific (*CXCR4*) genes in CD11b^+^ and CD11b^-^ cells isolated from human GBM (CD11b^+^ n = 13, CD11b^-^ n = 5), control brain (CD11b^+^ n = 5, CD11b^-^ n = 2) and blood monocyte samples (n = 2). Bar graphs illustrate the absolute number of transcripts normalized to 100,000 transcripts of *ACTB*. Analysis was done by students t test. Error bars indicate the SEM. *, p<0.05; **, p<0.01; ***, p<0.001

Next, we collected human glioma samples and isolated CD11b^+^ microglia/macrophages via MACS. We analyzed the gene expression of GAMs (CD11b^+^ sorted cells) in GBM samples, the flow through of these tumor samples (the CD11b-negative fraction after MACS-isolation), control microglia samples (taken from brain resections of hippocampus, epilepsy, and trauma patients), and blood monocytes (Fig. 6).

We detected high expression of all genes in CD11b^+^ GAMs isolated from GBM samples, however only the gene expression of *IL1RN, ISG20* (both M1-specific), and *TGFBI* (M2b-specific) was significantly higher in CD11b^+^ cells isolated from GBM samples, when compared to CD11b^+^ cells isolated from control brain samples. The expression of *CXCR4* was not significantly higher, whereas the expression of *CLEC7A* was significantly lower in CD11b^+^ cells isolated from GBM samples. This might be in part due to the fact that the control brain specimen were not taken from healthy patients, but patients suffering from epilepsy (3 samples) and trauma injury (1 sample). In these conditions the microglia might have already been polarized toward an M2-like phenotype.

### GAMs express the pro-tumorigenic genes *Gpnmb* and *Spp1*

Two features of high grade glioma are the aggressive invasion into the brain parenchyma and the immune-suppressive environment which prevents tumor rejection. Previously, we and other groups reported the expression of several pro-tumorigenic genes expressed in GAMs. We could confirm the expression of most of these genes in our dataset and a selection of these genes can be found in Table 4.

To identify novel glioma-regulated genes expressed in microglia/macrophages that might play a role in tumor-progression, we screened our dataset for genes that have been reported to play a pro-tumorigenic role in peripheral tumors, but have not been reported in GAMs. The genes *Gpnmb* and *Spp1* were two of the highest upregulated genes in our GAMs data set (Table 1), clustered into the glioma-regulated (red) module, and have been implicated in immune-suppression (*Gpnmb*) or tumor cell invasion (*Spp1*) in peripheral tumors. To investigate the expression of these genes in GAMs via qRT-PCR in FACS-sorted samples from GL261 and RCAS-PDGFb tumors in comparison to naïve control cells we used the same samples as described above for the validation of the M1 and M2a,b,c-specific genes.

**Fig 7:**
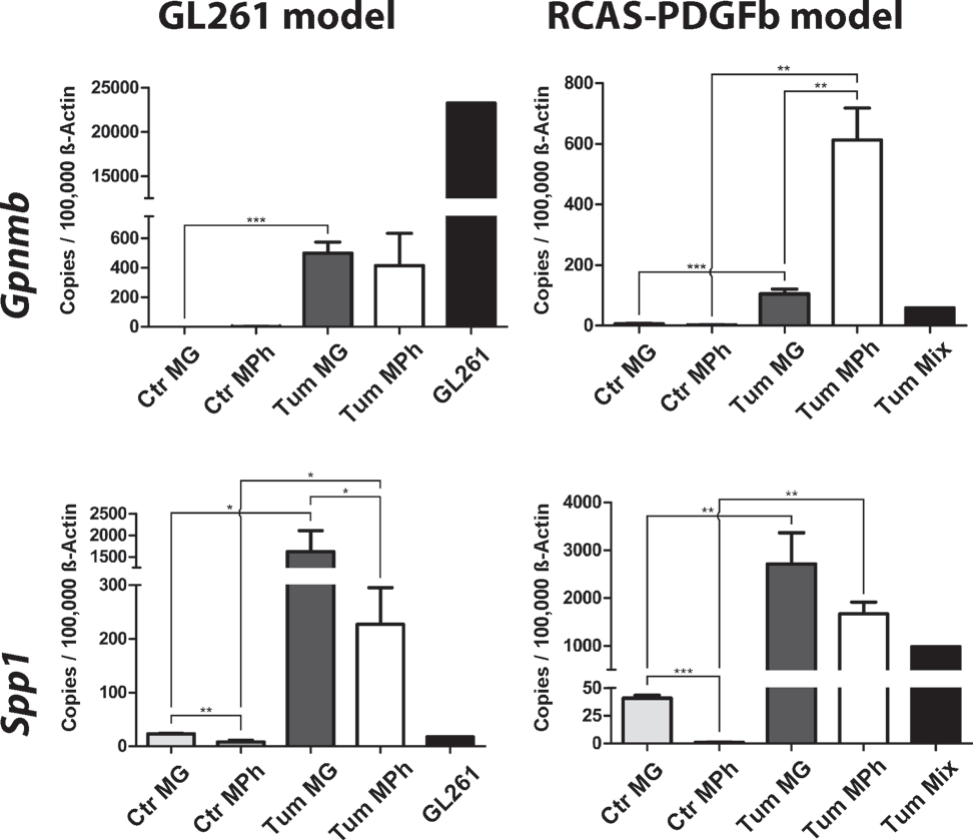
*Gpnmb* and *Spp1* expression is upregulated in murine GAMs. The gene expression of *Gpnmb* and *Spp1* was validated using qRT-PCR. For this we used the same FACS-sorted samples from GL261 and RCAS tumors as in Fig. 5. The upregulation of both genes could be confirmed in both tumor models. Resident microglia and invading macrophages/monocytes show different expression patterns of these genes. CTR MG: naïve microglia, CTR Mph: naïve monocytes, Tum MG: glioma-associated microglia, Tum Mph: glioma-associated macrophages/monocytes, GL261: cultured GL261 cells, Tum Mix: cultured RCAS-PDGFb tumor cells. Bar graphs illustrate the absolute number of transcripts normalized to 100,000 transcripts of *Actb* (n = 4). Analysis was done by students t test. Error bars indicate the Standard Error of Mean (SEM). *, p<0.05; **, p<0.01; ***, p<0.001

We were able to validate the expression of both genes using qRT-PCR. Furthermore, the regulation of the genes was similar in both brain tumor models – GL261, as well as RCAS-PDGFb tumors (Fig. 7). This shows that the up-regulation of these genes was not specific for one tumor model, but was observed in two different independent models. In addition, the expression of both genes was higher in GAMs when compared to the tumor cells (except for *Gpnmb* in the GL261 model), indicating that GAMs may be the primary source. Both genes were differently expressed in glioma-associated microglia and macrophages, as invading macrophages were the major source for *Gpnmb* in the RCAS-PDGFb model, whereas resident microglia were the main source for *Spp1* in both models. The expression level of *Gpnmb* in both, glioma-associated resident microglia and invading macrophages/monocytes, was similar in both glioma models. In contrast, the expression of *Spp1* was stronger in resident microglia and invading macrophages/monocytes isolated from RCAS-PDGFb tumors, when compared to the GL261 model.

To investigate the difference of gene expression in GAMs in the GL261 and the RCAS-PDGFb model in a broader set of genes we investigated the expression of six additional highly upregulated genes that clustered into the red or brown module in both models (S2 Fig.). From these six genes two were higher expressed in GAMs derived from the RCAS model (*CD300lf* and *Cd200r4*), two genes were expressed at similar levels in GAMs in both tumor models (*Trem1* and *Sh2d1b1*), and two genes were higher expressed in GAMs derived from the GL261 model (*Uck2* and *Creb5*).

### Expression of *GPNMB* and *SPP1* is upregulated in human GBM-associated microglia/macrophages

As described above, we collected human samples and isolated CD11b^+^ microglia/macrophages via MACS and used qRT-PCR to determine the expression of *GPNMB* and *SPP1* in these samples. In addition to CD11b^+^ and CD11b^-^ cells isolated from GBM samples and naïve brain tissue we also investigated meningioma and anaplastic astrocytoma (grade III glioma) samples.

**Fig 8:**
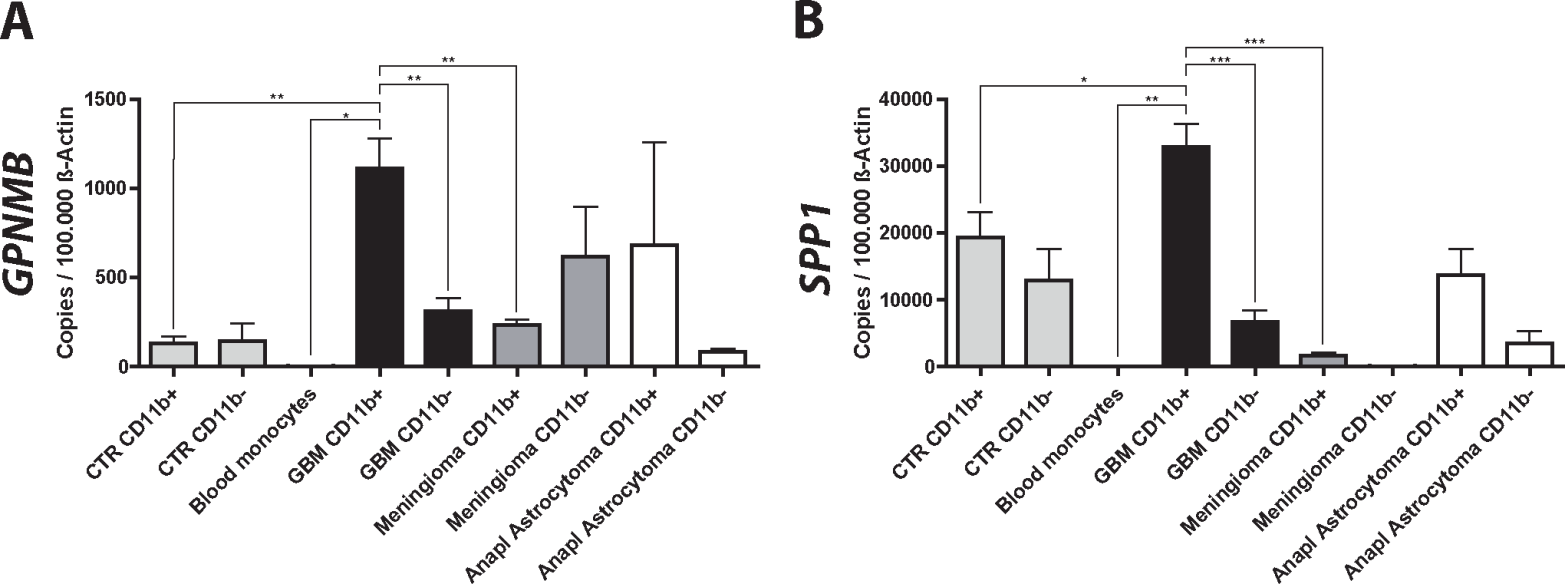
*GPNMB* and *SPP1* expression is upregulated in human GAMs. We determined the expression of the genes *GPNMB* and *SPP1* in CD11b^+^ and CD11b^-^ cells isolated from human GBM (CD11b^+^ n = 15, CD11b^-^ n = 9), meningioma (CD11b^+^ n = 5, CD11b^-^ n = 3), grade III anaplastic astrocytoma (CD11b^+^ n = 2, CD11b^-^ n = 2), control brain (CD11b^+^ n = 5, CD11b^-^ n = 2) and blood monocyte samples (n = 2). The expression of *GPNMB* and *SPP1* was significantly higher in CD11b^+^ cells isolated from GBMs compared to CD11b^+^ cells isolated from control brain, benign meningioma samples, blood monocytes, and CD11b^-^ cells in GBM. Bar graphs illustrate the absolute number of transcripts normalized to 100,000 transcripts of *ACTB*. Analysis was done by students t test. Error bars indicate the SEM. *, p<0.05; **, p<0.01; ***, p<0.001

We found that the expression of *GPNMB* and *SPP1* was significantly higher in human GAMs isolated from GBM samples, when compared to non-tumor-associated control microglia and blood monocytes (Fig. 8). Furthermore, the expression of these genes was significantly higher in GAMs than in the tumor flow through, indicating that GAMs are the predominant source for these transcripts (S3 Fig.). The source and rate of expression of *GPNMB* seems to be dependent on the grade of malignancy of the tumor. In GBM GAMs were the main source for the expression, showing a 5 times higher expression than the GBM flow through, which comprises tumor cells and other stromal cells. However, when looking at meningioma, a benign tumor of the meninges, the expression of *GPNMB* was higher in the CD11b^-^ cell fraction, compared to the CD11b^+^ cell fraction. The overall expression of *GPNMB* was higher in GBM than in meningioma and grade III astrocytoma samples. The expression level of *SPP1* was also dependent on tumor grade. Similar to *GPNMB*, the expression of *SPP1* was higher in GAMs isolated from GBM specimen, compared to the GBM flow through. Whereas, in lower grade tumors the expression of *SPP1* was generally lower, which might indicate a possible role for *SPP1* in higher grade tumors.

### High expression of *GPNMB* and *SPP1* in human GBM tissues is associated with poorer survival outcome

**Fig 9:**
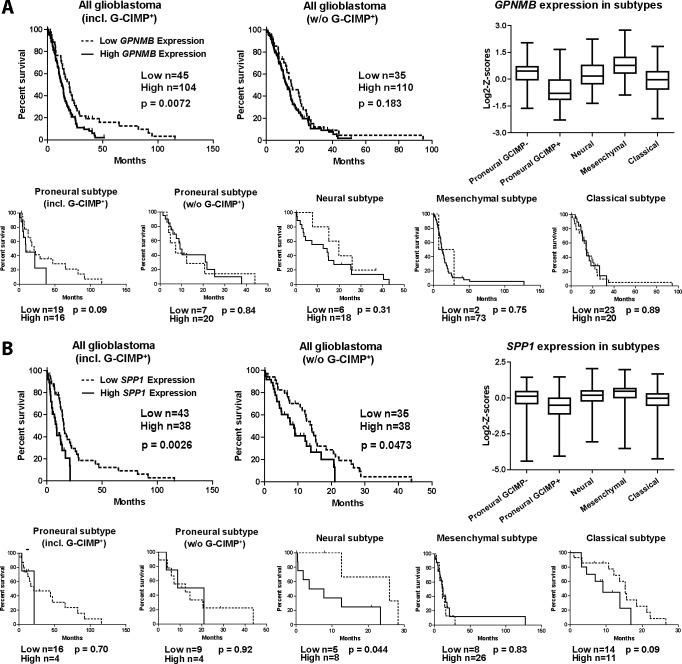
High *GPNMB* and *SPP1* expression is associated with worsened survival prognosis in human GBM patients. Data taken from the TCGA database, showing survival probability of glioma patients grouped according to high and low expression of our target genes *GPNMB* (A) and *SPP1* (B). High expression of both genes has a negative effect on patient prognosis. Patients were in addition grouped into the four molecular subtypes (proneural, neural, mesenchymal, and classical). Low *GPNMB* expression seems to have the most severe effect on patient prognosis in the proneural subtype when including G-CIMP^+^ tumors, but no significant effect in the other subtypes. Low *SPP1* expression seems to have the highest effect on patient prognosis in the neural and classical subtypes. Furthermore, both genes are differently regulated in the four subtypes (box plots in A and B) Significances for box plots: *GPNMB*: proneural GCIMP- (PG-) vs. proneural GCIMP+ (PG+) ***, PG- vs. mesenchymal (M) *** PG- vs. classical (C) **, PG+ vs. neural (N) ***, PG+ vs. M ***, PG+ vs. C **, N vs. M ***, N vs. C *, M vs. C ***. *SPP1*: PG- vs. M ***, PG+ vs. N ***, PG+ vs. M ***, C vs. M ***.*, p<0.05; **, p<0.01; ***, p<0.001

We used the cBioPortal database (http://www.cbioportal.org/public-portal/), to access TCGA data which links gene expression data to patient data, to investigate the effect of *GPNMB* and *SPP1* expression on patient prognosis [36,37]. We grouped patients into low and high expression (gene expression lower than the negative standard deviation or higher than the positive standard deviation, respectively) and determined the overall survival for these patients. High expression of each gene has a negative effect on patient prognosis. Median survival was 19.77 months (low *GPNMB* expression) vs. 12.92 months (high *GPNMB* expression) and 15.31 months (low *SPP1* expression) vs. 8.82 months (high *SPP1* expression) (Fig. 9). This dataset also included G-CIMP^+^ tumors that are mostly proneural subtype tumors and generally have a better overall survival prognosis when compared to G-CIMP^-^ tumors. We excluded these G-CIMP^+^ tumors from our analysis and reanalyzed the data. Most G-CIMP^+^ tumors exhibited a low expression of both, *GPNMB* and *SPP1*. Accordingly, the overall survival prediction for tumors with low expression of either *GPNMB* or *SPP1* was less favorable after removing G-CIMP^+^ tumors from the analysis. Median overall survival for GBM with low *SPP1* or *GPNMB* expression was 14.16 months (low *SPP1* expression including G-CIMP^+^ tumors) vs. 12.56 months (low *SPP1* expression excluding G-CIMP^+^ tumors) and 17.7 months (low *GPNMB* expression including G-CIMP^+^ tumors) vs. 14.82 months (low *GPNMB* expression excluding G-CIMP^+^ tumors). We also plotted the overall survival of patients with intermediate expression of each gene (S4 Fig.). Patients with high and intermediate *GPNMB* expression have a similar overall survival prognosis, whereas patients with low *GPNMB* expression have a significantly better prognosis. Median survival excluding G-CIMP^+^ tumors was 10.39 months (high *GPNMB* expression), 9.84 months (intermediate expression), and 14.82 months (low expression). In contrast, the survival prognosis of patients with intermediate *SPP1* expression is in between of patients with low and high *SPP1* expression. Median survival excluding G-CIMP^+^ tumors was 7.29 months (high *SPP1* expression), 10.49 months (intermediate expression), and 12.56 months (low expression).

In addition, we grouped patients into the four molecular subtypes (proneural incl. G-CIMP^+^ tumors and w/o G-CIMP^+^ tumors, neural, mesenchymal, and classical) to investigate the effect of high *GPNMB* and *SPP1* expression in these subtypes. Low *GPNMB* expression is associated with the most positive effect on patient prognosis in the proneural subtype if G-CIMP^+^ patients are included (p = 0.09, median survival of 20.66 months (low expression) vs. 9.21 months (high expression)), but not in the other subtypes. Low *SPP1* expression is associated with the most beneficial effect on patient prognosis in the neural (p = 0.044, median survival of 25.90 months vs. 5.65 months) and the classical subtype (p = 0.09, median survival of 15.31 months vs. 9.11 months). Furthermore, both genes are differently regulated within the four subtypes. Mesenchymal tumors have a higher probability of high *GPNMB* and *SPP1* expression, whereas classical tumors have a higher probability of low expression of both genes. G-CIMP^+^ tumors, which generally have a better survival prognosis, display the lowest expression of both genes, indicating a possible role of these genes in advanced malignancy.

To test for a proportional risk increase for survival with the gene expression level, we fitted a Cox proportional hazards regression model to our data including a test for the proportional hazards assumption. The expression level of both genes had a significant effect on survival of glioblastoma patients (all glioblastoma) when G-CIMP^+^ cases are included, as well as in the proneural subtype when G-CIMP^+^ cases are included. All data sets, except neural subtype tumors when tested for SPP1 expression, passed the test for the proportional hazards assumption (S5 Table).

## Discussion

Glioma-associated microglia/macrophages actively support glioma growth by the release of factors that stimulate angiogenesis, invasion, or suppression of immunity [9,10,11,15]. In the present study we performed a genome-wide expression analysis of these cells isolated from an experimental glioma mouse model. We have identified genes which are specifically regulated in glioma-associated microglia/macrophages when compared to naïve microglia or peripheral macrophages. We selected some of these genes and validated their expression in two independent glioma mouse models as well as in human glioma samples. In addition we could show that the expression of these genes differs in microglia and macrophages/monocytes. Our results are in accordance with previous reports that investigated the expression of selected target genes in GAMs via qRT-PCR [9,10,11], and markers known for tumor-associated macrophages in peripheral tumors. Several genes that are implicated in angiogenesis (*Vegfa, Hgf*), suppression of immunity (*Arg1, Tgfb3*), and tumor invasion (*Mmp2, Mmp14, Ctgf*) were also highly expressed in our GAMs data set (Table 4).

Previous studies reported that GAMs express both markers of the M1 and M2 macrophage phenotype [9,18]. However, no extensive comparison of these phenotypes has been performed yet. By comparing our expression data with those generated from M1, M2a, M2b, and M2c-polarized macrophages, we show that the GAM phenotype shows only partial overlap with the M1, M2a, M2b, and M2c phenotype. To date the term “M2-like polarization” has often been used for describing the polarization of GAMs; our data indicate that GAMs do not fit into a classical M1 or M2 phenotype, but represent a unique phenotype. As an alternate explanation it might reflect heterogeneity among GAMs. One might speculate that dependent on the location in the tumor tissue, some GAMs are polarized toward an M1-like phenotype, whereas other GAMs possess a more M2-like phenotype and another population of GAMs is not polarized toward M1 or M2-like states. Therefore, single-cell sequencing of GAMs would help to better understand the activation status of GAMs.

GAMs have been shown to promote tumor growth, rather than inhibiting it, e.g. by secreting factors that support glioma invasion or immunosuppressive factors [9,11,13,15]. Therefore, these cells represent an attractive target for anti-glioma therapy, as modulation of their activation state might be useful to inhibit glioma progression [14,16]. Here we present *GPNMB* and *SPP1* as possible new targets and could show that these genes are highly expressed in GAMs in different glioma mouse models, in human GBM, and that high expression of these genes is correlated with shorter glioma-patient survival. The expression of neither of these genes was previously linked to GAMs.

The expression of *GPNMB* has been reported in tumor cells for different cancers, including glioma [46,47]. Furthermore, *GPNMB* expression was detected in microglia of non-neoplastic rat brains and increased with inflammation [48]. The data from our GL261 glioma model suggests that GL261 cells express *GPNMB* at a very high level. However, in the RCAS-PDGFb glioma mouse model, as well as in human GBM samples GAMs were the predominant source for *GPNMB* expression in all tested paired samples (S3 Fig.). GPNMB (also called Osteoactivin) is a transmembrane protein, but is also localized in the phagosome and can also be secreted, and might have different functions in GAMs and in the tumor. Ripoll *et al*., have shown that GPNMB acts as a negative regulator of pro-inflammatory macrophage activation in RAW264.7 cells [49]. Thus, high *GPNMB* expression in GAMs could participate in the modulation of the pro-tumorigenic phenotype of GAMs. Furthermore, GPNMB has been shown to inhibit T cell activation via direct cell-cell interaction of antigen-presenting cells and T cells, and could thus contribute to the immunosuppressive milieu in gliomas [50,51,52]. Finally, anti-Gpnmb antibodies conjugated with a cytotoxic agent are under investigation for the treatment of malignant glioma, breast cancer, and cutaneous melanoma [53,54,55].

Furthermore, we found that GAMs highly express *SPP1*. SPP1 (also called Osteopontin) is a secreted protein that has been postulated to increase tumor cell invasion *in vivo* and migration *in vitro*, and was found to be highly expressed in different types of cancers, such as lung cancer, ovarian cancer, and also glioma [56,57,58,59,60,61]. Furthermore, SPP1 has recently been identified as a ligand for CD44. SPP1-CD44 interaction was shown to increase stemness of CD44-expressing glioma-initiating cells [62]. Here we show that GAMs and not other cells of the tumor microenvironment are the predominant source for *SPP1* expression in glioma.

Two studies have previously performed screens of freshly-isolated GAMs. We used these data sets to investigate the expression of *Gpnmb, Spp1, Il1rn, Isg20, Cxcr4, Tgfbi*, and *Clec7a* in these studies (S6 Table). Huang *et al*., performed a screen of mouse GFP^+^ chimeric GL261-associated and naïve (also isolated from the brain) bone-marrow-derived myeloid cells. However, using this approach they did not target the brain-resident microglia population that is also present in the tumor (Data set: E-GEOD-38283) [63]. All of the investigated genes, except *Cxcr4*, were also upregulated in our screen – however to a lesser degree. This was partially due to an already high expression in the control cells. Furthermore, Murat *et al*., performed a microarray experiment on a paired sample of human GAMs and whole tumor lysate from the same patient (Data set: GSE16119) [64]. All of the 7 genes were higher expressed in GAMs when compared to the whole tumor lysate. However, it should also be noted that the whole tumor lysate was not depleted of GAMs.

Taken together, our findings show that GAMs are polarized toward a phenotype that has only partial overlap with the M1 or M2a, M2b, and M2c phenotypes. Furthermore, we identified GAMs as the predominant source for the pro-tumorigenic proteins *GPNMB* and *SPP1* in murine and human malignant glioma – highlighting the importance of macrophages and microglia as therapeutic targets in anti-tumor treatment regimens.

## Supporting Information

S1 Fig GSEA analysis of the M1/M2a,b,c data sets against our GAMs data set. We performed GSEA analysis of >2 fold up- and downregulated genes in M1/M2a,b,c vs. M0 macrophages against the entire GAMs data set that resulted from the WGCNA analysis.
(TIF)

S2 Fig The expression levels of six additional glioma-regulated genes. The expression of *Cd300lf, Cd200r4, Trem1, Sh2d1b1, Creb5*, and *Uck2* in flow-sorted glioma-associated microglia and macrophages/monocytes isolated from mouse GL261 and RCAS-PDGFb tumors.
(TIF)

S3 Fig *GPNMB* and *SPP1* expression in CD11b^+^ and paired CD11b^-^ fractions isolated from human GBM samples. (TIF)

S4 Fig Kaplan-Meier curves of TCGA data of patients with high, intermediate, and low *GPNMB* or *SPP1* expression. (TIF)

S1 Table Primer sequences that were used for mouse and human qRT-PCRs and generation of nested amplicons. (TXT)

S2 Table List of all significantly up- and downregulated genes. (XLSX)

S3 Table Results of the WGCNA analysis. A list of all 10,875 genes that were used for WGCNA analysis and the corresponding modules they were clustered into.
(XLSX)

S4 Table All genes that were included in the overrepresented GO terms of the GAMs set. (XLSX)

S5 Table Results of the Cox proportional hazards regression model of the TCGA data for *GPNMB* and *SPP1*. (DOCX)

S6 Table Comparison with other screens on GAMs. The expression values of *Gpnmb, Spp1, Il1rn, Isg20, Clec7a, Tgfbi*, and *Cxcr4* in our microarray screen and in two studies that performed microarrays on GAMs are listed [63,64].
(DOCX)
