# Supplementary figures and images for: Glioma-Associated Microglia/Macrophages Display an Expression Profile Different from M1 and M2 Polarization and Highly Express Gpnmb and Spp1
Source: PLoS One. 2015 Feb 6;10(2):e0116644. doi: 10.1371/journal.pone.0116644 (PMC4320099; doi:10.1371/journal.pone.0116644)

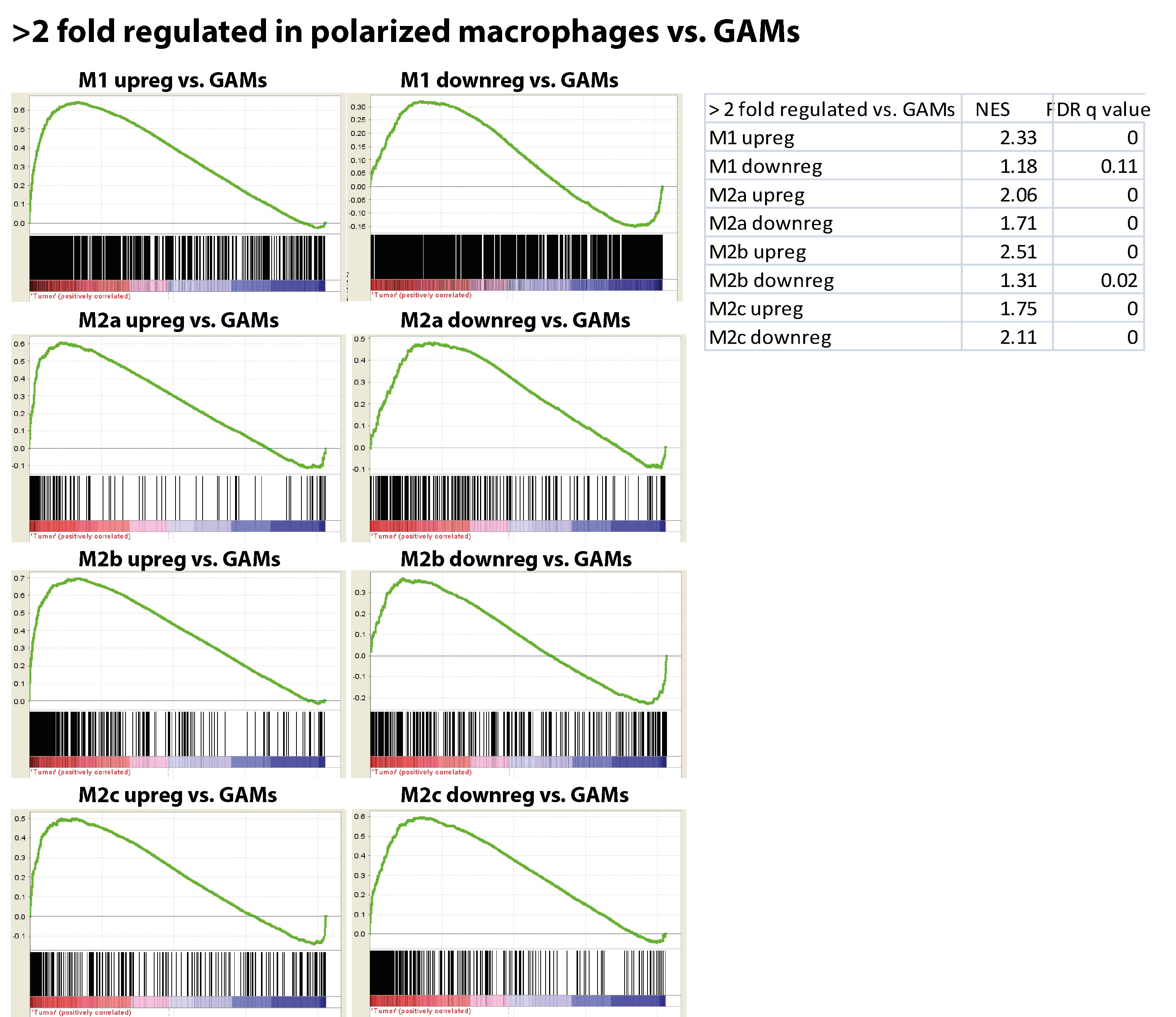

Supplement: S1 Fig — We performed GSEA analysis of >2 fold up- and downregulated genes in M1/M2a,b,c vs. M0 macrophages against the entire GAMs data set that resulted from the WGCNA analysis. (TIF) [file pone.0116644.s001.tif]

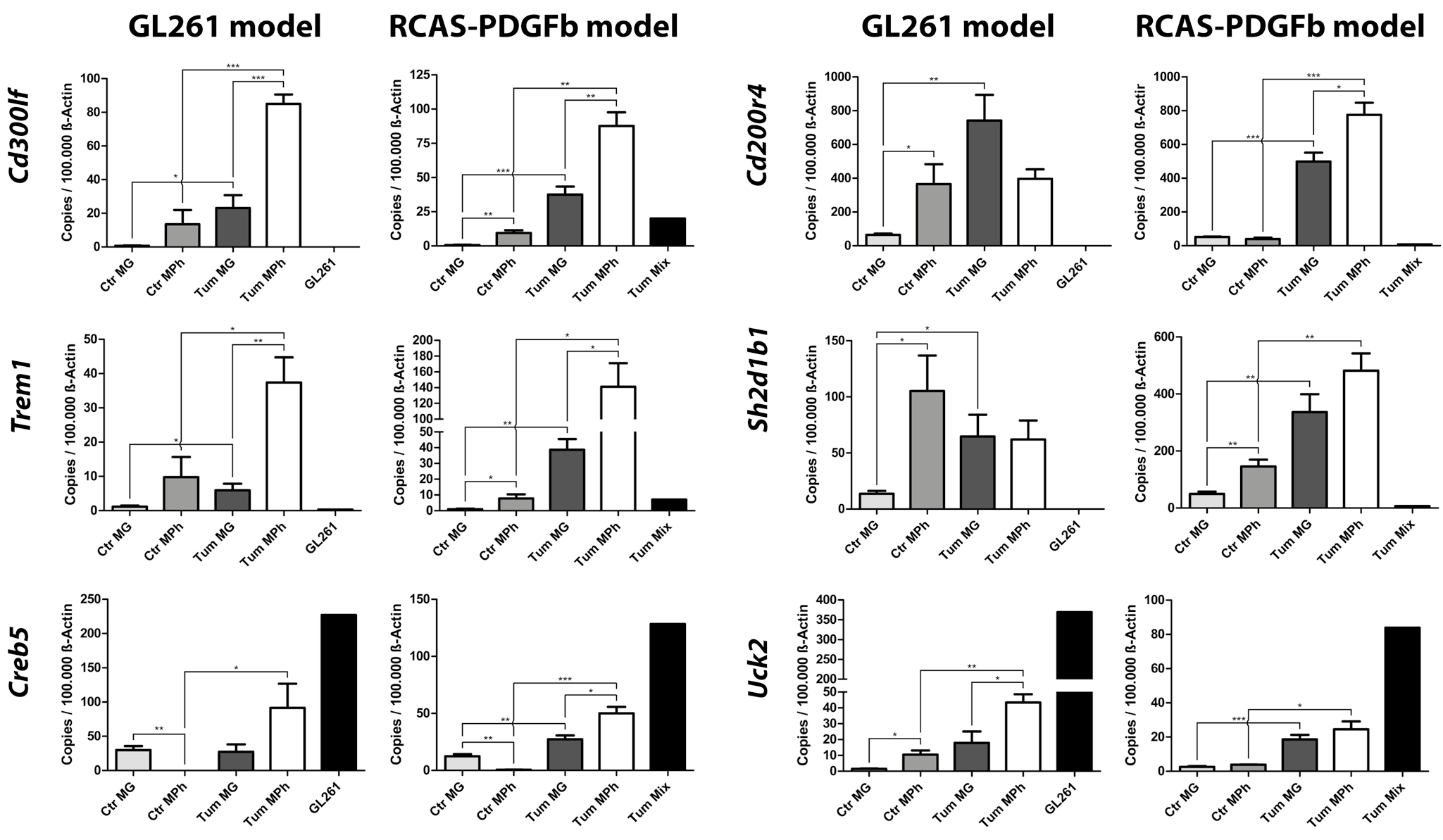

Supplement: S2 Fig — The expression of Cd300lf, Cd200r4, Trem1, Sh2d1b1, Creb5, and Uck2 in flow-sorted glioma-associated microglia and macrophages/monocytes isolated from mouse GL261 and RCAS-PDGFb tumors. (TIF) [file pone.0116644.s002.tif]

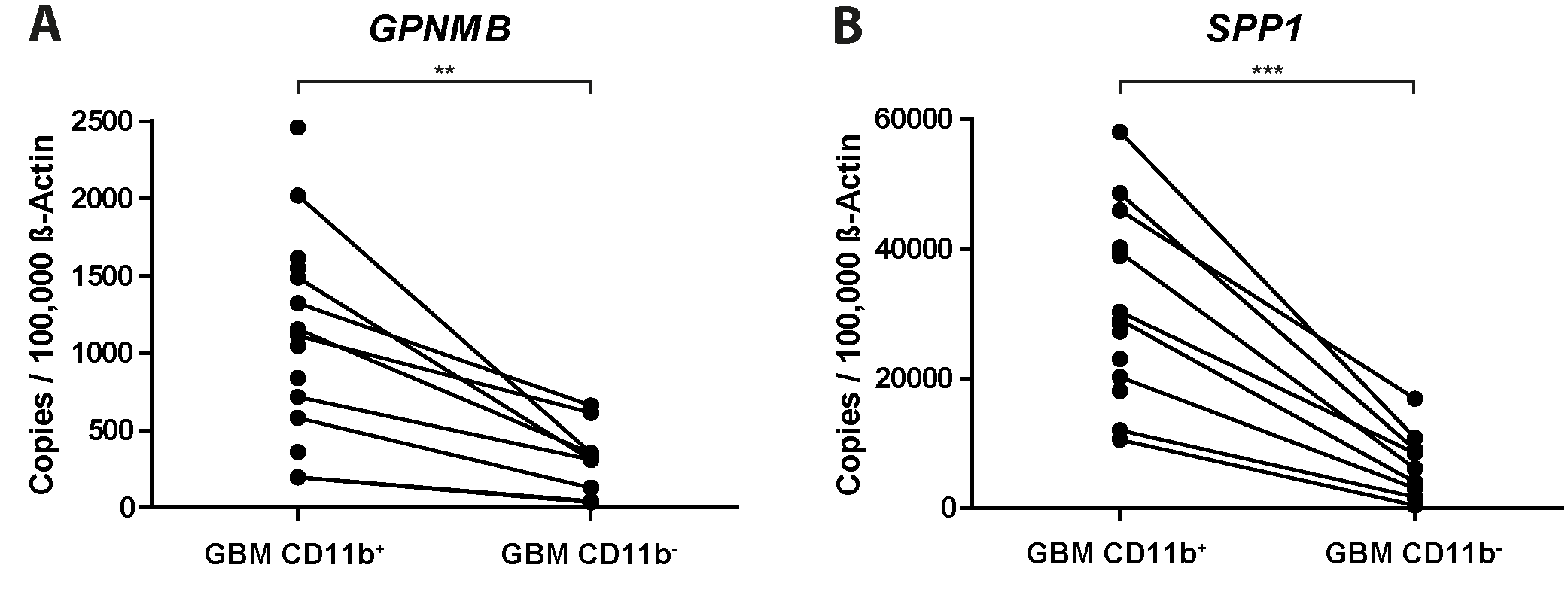

Supplement: S3 Fig — (TIF) [file pone.0116644.s003.tif]

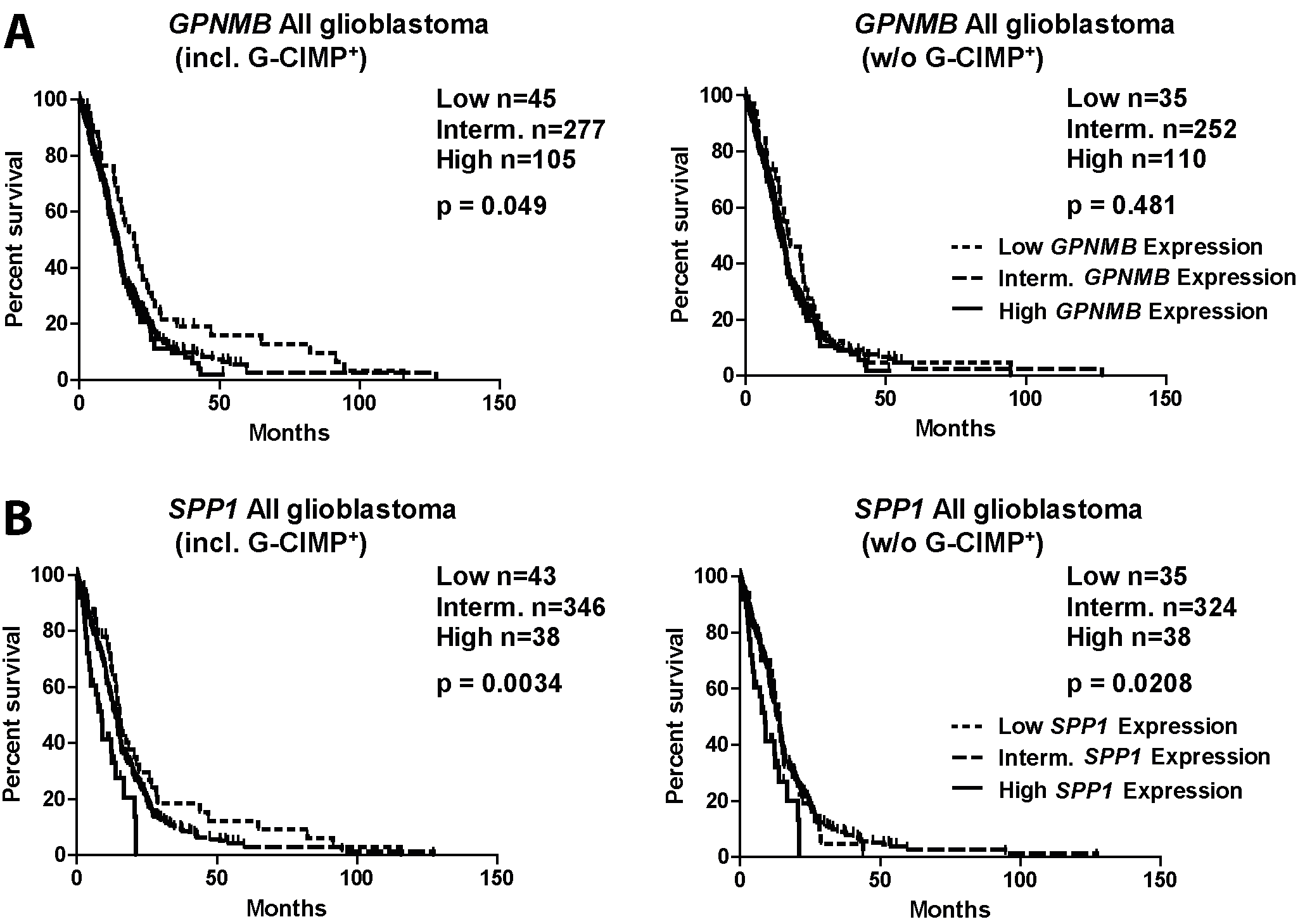

Supplement: S4 Fig — (TIF) [file pone.0116644.s004.tif]
