# Supplementary material for: Glioma-Associated Microglia/Macrophages Display an Expression Profile Different from M1 and M2 Polarization and Highly Express Gpnmb and Spp1
Source: PLoS One. 2015 Feb 6;10(2):e0116644. doi: 10.1371/journal.pone.0116644 (PMC4320099; doi:10.1371/journal.pone.0116644)
Supplement: S5 Table — (DOCX) [file pone.0116644.s009.docx]

|  | ***GPNMB*** | | | ***SPP1*** | | |
| --- | --- | --- | --- | --- | --- | --- |
| Patient group | p value  Proportional Hazards  Assumption | Hazard Ratio | p value  Hazard Ratio | p value  Proportional Hazards  Assumption | Hazard Ratio | p value  Hazard Ratio |
| All glioblastoma (incl. G-CIMP) | 0.828 | 1.165 [1.035-1.313] | 0.01162 | 0.270 | 1.200 [1.060-1.359] | 0.00259 |
| All glioblastoma (w/o G-CIMP) | 0.572 | 1.066 [0.934-1.218] | 0.34246 | 0.067 | 1.102 [0.962-1.261] | 0.15015 |
| Proneural subtype (incl. G-CIMP) | 0.694 | 1.465 [1.132-1.896] | 0.00317 | 0.599 | 1.444 [1.129-1.846] | 0.00111 |
| Proneural subtype (w/o G-CIMP) | 0.693 | 1.175 [0.821-1.680] | 0.37559 | 0.725 | 1.219 [0.948-1.568] | 0.09251 |
| Neural subtype | 0.305 | 1.075 [0.791-1.460] | 0.64585 | 0.021 | 1.187 [0.786-1.793] | 0.41700 |
| Mesenchymal subtype | 0.601 | 0.946 [0.711-1.257] | 0.70051 | 0.265 | 1.042 [0.783-1.386] | 0.77631 |
| Classical subtype | 0.456 | 1.071 [0.793-1.447] | 0.65242 | 0.363 | 1.000 [0.759-1.317] | 0.99781 |
