# Supplementary material for: Glioma-Associated Microglia/Macrophages Display an Expression Profile Different from M1 and M2 Polarization and Highly Express Gpnmb and Spp1
Source: PLoS One. 2015 Feb 6;10(2):e0116644. doi: 10.1371/journal.pone.0116644 (PMC4320099; doi:10.1371/journal.pone.0116644)
Supplement: S6 Table — The expression values of Gpnmb, Spp1, Il1rn, Isg20, Clec7a, Tgfbi, and Cxcr4 in our microarray screen and in two studies that performed microarrays on GAMs are listed [63,64]. (DOCX) [file pone.0116644.s010.docx]

|  | This study | Huang et al. 2014 | Murat et al. 2009 |
| --- | --- | --- | --- |
|  | mouse GL261-associated CD11b^+^ microglia/macrophages vs. naïve microglia and peritoneal macrophages | mouse GL261-associated bone marrow-derived vs. naive bone-marrow | human GAMs vs. matched whole tumor extract (1 patient) |
| *Gpnmb* | 36 fold upreg. | 2.7 fold upreg. | 1.3 fold upreg. |
| *Spp1* | 27.2 fold upreg. | 2.3 fold upreg. | 18.4 fold upreg. |
| *Il1rn* | 14.8 fold upreg. | 1.9 fold upreg. | 3.3 fold upreg. |
| *Isg20* | 3.4 fold upreg. | 1.5 fold upreg. | 2.3 fold upreg. |
| *Clec7a* | 4.7 fold upreg. | 1.4 fold upreg. | 9.8 fold upreg. |
| *Tgfbi* | 3.3 fold upreg. | 1.1 fold upreg. | 16.1 fold upreg. |
| *Cxcr4* | 10.6 fold upreg. | 1.1 fold downreg. | 3.5 fold upreg. |
